# Deciphering associations between gut microbiota and clinical factors using microbial modules

**DOI:** 10.1093/bioinformatics/btad213

**Published:** 2023-04-21

**Authors:** Ran Wang, Xubin Zheng, Fangda Song, Man Hon Wong, Kwong Sak Leung, Lixin Cheng

**Affiliations:** Shenzhen People's Hospital, First Affiliated Hospital of Southern University of Science and Technology, Second Clinical Medicine College of Jinan University, Shenzhen 518020, China; Department of Computer Science and Engineering, The Chinese University of Hong Kong, Shatin, New Territories, Hong Kong; Shenzhen People's Hospital, First Affiliated Hospital of Southern University of Science and Technology, Second Clinical Medicine College of Jinan University, Shenzhen 518020, China; Department of Computer Science and Engineering, The Chinese University of Hong Kong, Shatin, New Territories, Hong Kong; School of Data Science, The Chinese University of Hong Kong, Shenzhen 518000, China; Department of Computer Science and Engineering, The Chinese University of Hong Kong, Shatin, New Territories, Hong Kong; Department of Computer Science and Engineering, The Chinese University of Hong Kong, Shatin, New Territories, Hong Kong; Department of Applied Data Science, Hong Kong Shue Yan University, North Point, Hong Kong; Shenzhen People's Hospital, First Affiliated Hospital of Southern University of Science and Technology, Second Clinical Medicine College of Jinan University, Shenzhen 518020, China; Guangdong Provincial Clinical Research Center for Geriatrics, Shenzhen Clinical Research Center for Geriatrics, Shenzhen 518020, China

## Abstract

**Motivation:**

Human gut microbiota plays a vital role in maintaining body health. The dysbiosis of gut microbiota is associated with a variety of diseases. It is critical to uncover the associations between gut microbiota and disease states as well as other intrinsic or environmental factors. However, inferring alterations of individual microbial taxa based on relative abundance data likely leads to false associations and conflicting discoveries in different studies. Moreover, the effects of underlying factors and microbe–microbe interactions could lead to the alteration of larger sets of taxa. It might be more robust to investigate gut microbiota using groups of related taxa instead of the composition of individual taxa.

**Results:**

We proposed a novel method to identify underlying microbial modules, i.e. groups of taxa with similar abundance patterns affected by a common latent factor, from longitudinal gut microbiota and applied it to inflammatory bowel disease (IBD). The identified modules demonstrated closer intragroup relationships, indicating potential microbe–microbe interactions and influences of underlying factors. Associations between the modules and several clinical factors were investigated, especially disease states. The IBD-associated modules performed better in stratifying the subjects compared with the relative abundance of individual taxa. The modules were further validated in external cohorts, demonstrating the efficacy of the proposed method in identifying general and robust microbial modules. The study reveals the benefit of considering the ecological effects in gut microbiota analysis and the great promise of linking clinical factors with underlying microbial modules.

**Availability and implementation:**

https://github.com/rwang-z/microbial_module.git.

## 1 Introduction

The human gut harbors more than 100 trillion microorganisms, composing the most complex and abundant ecosystem, referred to as human gut microbiota (Turnbaugh et al. 2007; Belizário and Napolitano 2015). Microorganisms play a vital role in maintaining body health by helping to digest foods, producing indispensable metabolites and hormones, training the immune system, protecting against colonization of pathogens, participating in drug metabolism, and contributing to more significant systemic effects on mental health through the “gut-brain axis” (Foster and Neufeld 2013; Vázquez-Baeza et al. 2018). It is reported that a collection of bacterial species is shared by healthy adults (Lozupone et al. 2012), and the composition of healthy individuals is relatively stable in the absence of interventions (Halfvarson et al. 2017). Shifting away of the gut microbiota from its healthy state, which is called dysbiosis, is associated with diverse diseases, e.g. obesity (Lozupone et al. 2012), cardiovascular diseases (Jie et al. 2017; Liu et al. 2020), cancers (Gopalakrishnan et al. 2018), major depression (Cheung et al. 2019), inflammatory bowel disease (IBD; Durack and Lynch 2019), etc. Dysbiosis includes the alterations in both taxonomic composition, e.g. elevated or decreased relative abundance of particular taxa and changed diversity of the whole microbial community (Belizário and Napolitano 2015; Gilbert et al. 2016), and temporal dynamics, where the variation of gut microbiota over time in some conditions are discovered more pronounced compared with the healthy controls (Halfvarson et al. 2017, Schirmer et al. 2018, Lloyd-Price et al. 2019; Zhou et al. 2019).

Associations between gut microbiota and various diseases have been identified by numerous metagenome-wide association studies (Gilbert et al. 2016; Halfvarson et al. 2017; Franzosa et al. 2019; Zhou et al. 2019; Dan et al. 2020). However, most of these studies focus on linking individual microbial taxa with disease states, and there are some limitations. Firstly, the measured gut microbiota is generally in the form of compositional data, represented as the relative abundance of each taxon in the whole community, where the captured abundance of a taxon depends on that of the others. It would be flawed to infer the expansion or depletion of a single taxon based on the relative abundance since the growth/loss of a taxon could actually be the decrease/rise of the others, which might lead to false associations and conflicting discoveries in different studies (Gilbert et al. 2016; Duvallet et al. 2017; Wirbel et al. 2019; Nearing et al. 2022). Secondly, instead of alterations of a few taxa, gut microbial changes in some conditions involve a larger set of taxa or a broad restructuring of the community, especially IBD, where the investigation of groups of related taxa is crucial (Duvallet et al. 2017; Gilbert et al. 2018; Durack and Lynch 2019). In addition, the gut microbiota is a complex ecology where the metabolic interactions and resource competitions amongst the microorganisms are essential for community stability and host health (Wegener et al. 2011). Changes in the abundance of a taxon may also shape that of the others, resulting in correlated abundance over time and co-occurrence in different communities. Therefore, it might be more robust to investigate the alterations of human gut microbiota using microbial groups of related taxa compared with individual taxa (Lozupone et al. 2012; Cheng et al. 2019).

In addition to disease states, the gut microbiota is influenced by a variety of host-intrinsic, microbial, and environmental factors, including genetics, age, medication use, diet, lifestyle, body mass index, etc. (Gilbert et al. 2018; Schmidt et al. 2018). It is critical to uncover the associations of not only disease states but also other clinical factors with the gut microbiota in both taxonomic composition and temporal dynamics to understand the inherent mechanisms and to develop potential interventions for preventive or therapeutic purposes (Gilbert et al. 2018). However, commonly examined clinical factors only explain a tiny proportion of the interindividual diversity, leaving many underlying factors uncovered (Lloyd-Price et al. 2019). Taking all these considerations into account, we reason that multiple underlying factors collectively determine the taxonomic composition and temporal dynamics of gut microbiota through both direct regulations and indirect influences via ecological microbe–microbe interactions. The taxa affected by the same factor would have similar underlying abundance patterns and are thereby defined as a microbial module. The composition and dynamics of gut microbiota are observed outcomes of the underlying patterns.

In this study, we proposed a novel method to identify the underlying microbial modules, i.e. groups of taxa with similar abundance patterns affected by a common latent factor, from human longitudinal gut microbiota based on tensor factorization, which is a method that enables uncovering the underlying structure and latent factors from noisy data (Kolda and Bader 2009, Papalexakis et al. 2016). Specifically, the longitudinal data at the species level was modeled as a third-order tensor, and the compositional relative abundance was factorized into the contributions of microbial modules. On account of that the gut microbial composition of a subject should change smoothly over time, we modelled correlated activities of the microbial modules at neighbor time points (visits) in the method. As identified from longitudinal data, the modules reflected both the compositional and dynamic characteristics of the gut microbiota. We applied the method to IBD, a group of intestinal disorders characterized by chronic inflammation of the gastrointestinal tract and imbalances between microbes and immune systems (Schirmer et al. 2018; Lloyd-Price et al. 2019). The microbial modules identified from the cohort comprised of IBD patients and controls were further analyzed in terms of interindividual variation, intramodule taxa relationships, and associations with clinical factors, especially disease states, and were validated in external cohorts.

## 2 Materials and methods

### 2.1 Longitudinal gut microbiota data

**Figure 1:**
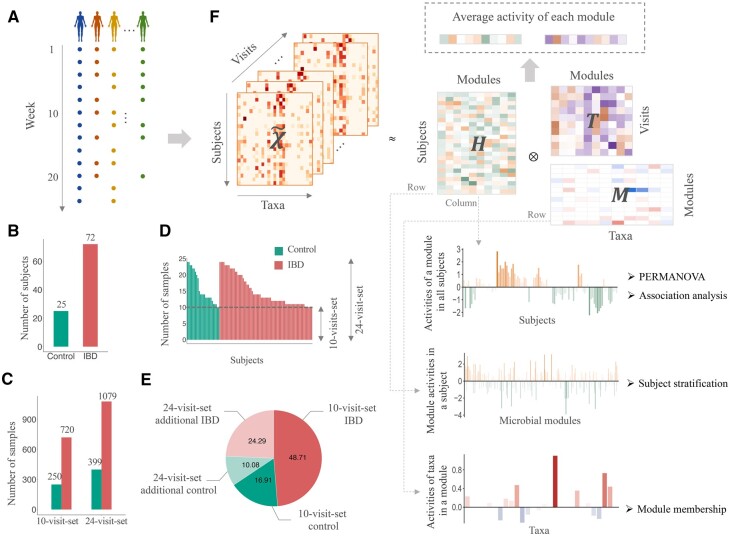
Overview of the study. (A) Fecal samples were collected consecutively from IBD patients and healthy controls for 1 year. (B) Number of healthy controls and IBD patients included in the study. (C) Number of control and IBD samples in the *“10-visit-set”* and *“24-visit-set”*. (D) Number of samples collected from each subject. E. Pie chart of the distribution of samples collected from healthy controls and IBD patients in the *“10-visit-set”* and *“24-visit-set”* additionally. The samples in the *“10-visit-set”* are also included in the *“24-visit-set”*. (F) Illustration of microbial module identification and downstream analysis in this study. The longitudinal data are represented as a third-order tensor and factorized into three factor matrices, which are further used for module analysis, subject stratification, and module member detection, respectively. PERMANOVA, permutational multivariate analysis of variance.

The human longitudinal gut microbiota data that captured the taxonomic composition of the gut microbial community at different time points (visits) were collected. The discovery set of the study was collected from the integrative Human Microbiome Project et al. (2019), the second phase of the Human Microbiome Project (HMP; Turnbaugh et al. 2007). Fecal samples were collected consecutively from the IBD patients (*n *=* *103) and healthy controls (*n *=* *27) for 1 year (Fig. 1A) and sequenced by shotgun metagenomic sequencing. The highest sampling frequency was every 2 weeks, resulting in 1638 samples with up to 24 samples for each subject. The taxonomic profile, functional profile, and corresponding metadata were downloaded from the Inflammatory Bowel Disease Multi’omics Database (https://www.ibdmdb.org). Quality control of the data was done by KneadData (https://huttenhower.sph.harvard.edu/kneaddata/). The taxonomic and functional profiles were generated using MetaPhlAn2 (Truong et al. 2015) and HUMAnN2 (Franzosa et al. 2018), respectively. Subjects with less than 10 samples were removed. As a result, 97 subjects were included for further analysis, consisting of 25 controls and 72 IBD patients [47 Crohn's disease (CD) and 25 ulcerative colitis (UC)]. We focused on the species level in this study (virus excluded) and explored the prevalent species. The species with nonzero abundance in less than 10% of samples were filtered out, leaving 123 species of interest. All samples from the 96 subjects comprised the dataset *“24-visit-set”*, including 1079 IBD samples and 399 control samples. Since the number of samples differed among the subjects with various sampling intervals, we generated a subset where 10 samples with roughly equal sampling intervals were selected for each subject, resulting in 720 IBD samples and 250 control samples, which was labeled as the *“10-visit-set”* (Fig. 1B–E and Table 1).

**Table 1.** Summary of the datasets used in the study.

Dataset	24-visit set	10-visit set	Val_Hall	Val_Lewis	Val_HHS
Data source	iHMP		Hall et al. (2017)	Lewis et al. (2015)	HMP HHS
		
Taxa (species)	123		81	79	123
IBD subjects	72		18	31	0
Control subjects	25		12	0	54
Samples of each subject	≤ 24	10	≤ 12	≤ 4	3
IBD samples	1079	720	162	99	0
Control samples	399	250	73	0	162
Completeness	Incomplete	Complete	Incomplete	Incomplete	Complete
Constructed tensor	$\hat{\chi }$	$\tilde{\chi }$	$\chi^{\prime }$		
Factorization result	24-visit (uncorrelated)	10-visit (uncorrelated)	Val_Hall-res (uncorrelated)		
		10-visit correlated			

Three external validation cohorts were collected and named as *“Val_Hall”* (Hall et al. 2017), *“Val_Lewis”* (Lewis et al. 2015), and *“Val_HHS”* (Lloyd-Price et al. 2017), respectively. *“Val_Hall”* consisted of both IBD and control subjects, whereas *“Val_Lewis”* and *“Val_HHS”* included only IBD subjects and only healthy subjects, respectively. The samples in *“Val_Hall”* were collected monthly from 18 IBD (9 CD and 9 UC) patients and 12 controls, up to 12 samples for each subject and 235 samples in total (Supplementary Fig. S1A–C), which were available at NCBI SRA under the BioProject PRJNA385949. We selected the samples without using antibiotics provided in Lewis et al. (2015; available at NCBI SRA, accession number: SRP057027) to construct the validation set *“Val_Lewis”*, resulting in 99 samples of 31 CD patients in total and up to 4 samples for each subject. Although healthy control samples were provided in Lewis et al. (2015) as well, they were one-visit data rather than longitudinal data, so that they were not used in the validation set. The samples of *“Val_Hall”* and *“Val_Lewis”* were downloaded from NCBI SRA in the format of FASTQ files using the SRA toolkit (https://trace.ncbi.nlm.nih.gov/Traces/sra/sra.cgi?view=software) and then processed following the bioBakery workflow as the discovery set (McIver et al. 2018). For *“Val_HHS”*, we downloaded the preprocessed taxonomic profile (generated by MetaPhlAn2 after quality control using KneadData) from the healthy human subjects (HHS) study (available at https://www.hmpdacc.org/hmsmcp2/) of HMP (Lloyd-Price et al. 2017) and excluded the subjects with less than three samples. The taxonomic profiles of the three validation sets were filtered by the species of interest in the discovery set, leaving 81, 79, and 123 species in *“Val_Hall”*, *“Val_Lewis”*, and *“Val_HHS”*, respectively (Table 1). The discovery and validation sets were preprocessed using the same workflow.

### 2.2 Identification of gut microbial modules

The longitudinal data of human gut microbiota was modeled as a third-order tensor $\chi \in R^{N\times L\times K}$, where N, L, and K were the numbers of subjects, taxa and visits (time points), respectively. The entry $\chi_{\mathrm{nlk}}$ represented the relative abundance of taxon $l\in \left\{1,\ldots ,L\right\}$ in the sample collected from subject $n\in \left\{1,\ldots ,N\right\}$ at visit $k\in \left\{1,\ldots ,K\right\}$. The horizontal slice $\chi_{n,.,.}$ consisted of the samples collected from subject n at different time points. The *“10-visit-set”* including 10 samples for each subject constructed a complete tensor $\tilde{\chi }\in R^{97\times 123\times 10}$, where N=97, L=123, and K=10.

**Figure 2:**
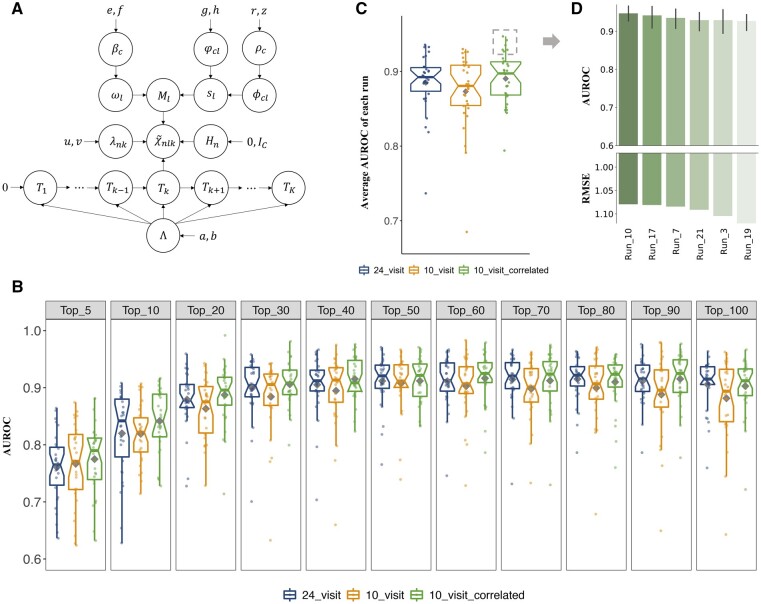
Decomposition results of the longitudinal gut microbiota data. (A) The Bayesian tensor factorization model of the proposed method (*visit-correlated model*). (B) Classification performance (AUROC) of different numbers of top-ranking microbial modules of IBD identified from the results of *“24_visit”*, *“10_visit”*, and *“10_visit-correlated”*, respectively. Dots indicate the performance of different random runs. Average values are demonstrated as grey diamonds. (C) Boxplot of the average AUROC of each run under different settings. The best performing runs of *“10_visit-correlated”* are marked in the grey dashed rectangle. (D) Comparison of the best performing runs of *“10_visit-correlated”* in terms of the classification performance and reconstruction error measured by the average AUROC using different numbers of top-ranking modules and RMSE, respectively.

To identify the underlying microbial modules that collectively contributed to the structure of the microbial community, the relative abundance tensor $\tilde{\chi }$ was decomposed into three factor matrices in terms of subjects ($H\in R^{N\times C}$), taxa ($M\in R^{C\times L}$), and visits ($T\in R^{K\times C}$), respectively, which shared a common dimension of C microbial modules (Fig. 1F). Each row of M represented a microbial module where $M_{cl}$ indicated the activity of microbial taxon l in module c. The factor matrices H and T represented the activities of the microbial modules in the subjects and visits, respectively. $H_{\mathrm{nc}}$ indicated the contribution of module c to the gut microbiota of subject n. $T_{.c}$ indicated the trend of module c over time. Specifically, the problem was modeled as Bayesian tensor factorization (Fig. 2A): where $c\in \left\{1,\ldots ,C\right\}$, C was the number of underlying microbial modules and $C\leq C_{\max}$. $C_{\max}$ was the predefined maximum number of microbial modules. $\theta =\left\{H,\;M,\;T,\ldots \right\}$ was the set of all variables in the model. $\lambda \in R^{N\times K}$ represented the precision where the same term $\lambda_{nk}$ was shared by the taxa for visit t of subject n, modeling the noise in the data.


(1) $$P\left(\left.\tilde{\chi }\right|\theta \right)=\prod_{nlk}𝒩\left({\tilde{\chi }}_{nlk}|\sum_{c}H_{nc}M_{cl}T_{kc},{\lambda_{nk}}^{-1}\right),$$

(2) $$\lambda_{nk}∼\mathrm{Gamma}\left(u,\;v\right),$$

It has been discovered in previous studies that sparsity on the factor matrices could induce clustering to produce coherent groups (Papalexakis et al. 2012). In order to identify sparse microbial modules and to group taxa with similar abundance patterns together, we imposed the spike-and-slab prior (Do et al. 2006; Titsias and Lázaro-Gredilla 2011; Hore et al. 2016) on the factor matrix M for sparse structure (Supplementary Methods): where $p_{cl}$ was the mixing weight between the Gaussian and the point mass, $\beta_{c}$ was the precision of the Gaussian distribution and $\delta_{0}(\cdot )$ denoted the Dirac delta function centered at zero. For efficient inference of the model, it was reparameterized as follows (Titsias and Lázaro-Gredilla 2011):


(3) $$M_{cl}∼p_{cl}N\left(M_{cl}|0,{\beta_{c}}^{-1}\right)+\left(1-p_{cl}\right)\delta_{0}\left(M_{cl}\right),$$

(4) $$M_{cl}=\omega_{cl}s_{cl},$$

(5) $$\omega_{cl}∼N\left(\omega_{cl}|0,{\beta_{c}}^{-1}\right),$$

(6) $$s_{cl}∼\mathrm{Bernoulli}\left(s_{cl}|p_{cl}\right),$$

(7) $$p_{cl}=\varphi_{cl}\phi_{cl}.$$

Additionally, given a limit of the number of modules $C_{\max}$, the spike-and-slab prior could also automatically determine the model complexity $C\leq C_{\max}$, i.e. the number of modules underlying the data, by generating C nonzero vectors in each factor matrix.

In the longitudinal gut microbiota studies, multiple samples were collected from the same subject at different visits. The repeated measures of the same subject were expected to be correlated (Chen and Li 2016). To model the correlations, we assumed that the activity of a microbial module c at time point k was dependent on that at the previous time point k-1: where $c\in \left\{1,\ldots ,C\right\}$, $k=\left\{2,\ldots ,K\right\}$ and $\Lambda_{c}$ was the precision shared by $T_{.c}$. The activities of the microbial modules in the subjects were assumed to be independent and follow the Gaussian distribution $H_{n.}∼N_{C}\left(0,I_{C}\right)$. The above model accounting for the correlations between samples from the same subject was referred to as the *visit-correlated model* in this article. The model was inferred using Variational Bayes (Jordan et al. 1999). See Supplementary Methods for the full Bayesian model, the update rules for the variables, and more details of the method.


(8) $$T_{kc}∼N\left(T_{k-1,c},{\Lambda_{c}}^{-1}\;\right),$$

(9) $$T_{1,c}∼N\left(0,{\Lambda_{c}}^{-1}\right),$$

(10) $$\Lambda_{c}∼\mathrm{Gamma}\left(a,b\right),$$

The members of a module were determined by the posterior inclusion probabilities (PIP), calculated as $E_{Q}\left(s_{cl}\right)$, which indicated the probability that a variable was included in the true model (Hore et al. 2016). A taxon was determined to be a member of a module if the corresponding PIP>0.5. Distinct initializations of the variables would lead to different factorization results. To find a better result and to conduct a fair evaluation of the overall performance of the model, 30 runs with random initializations were executed and compared in two aspects: (i) the classification performance using the top-ranking modules (see *Classification of the subjects*), and (ii) the reconstruction error measured by the root mean square error (RMSE) between the original tensor $\tilde{\chi }$ and the reconstructed tensor $\tilde{\chi }\prime =H⊗M⊗T$. In comparison to the visit-correlated model, we also implemented a model that did not account for the correlations between the samples collected from the same subject, referred to as the *visit-uncorrelated model* (Supplementary Fig. S2 and Supplementary Methods).

### 2.3 Analysis of microbial modules

The variations of taxonomic composition (intersample) and the microbial module activities (interindividual) explained by several clinical factors were quantified via permutational multivariate analysis of variance (PERMANOVA) using the adonis function in the R package “Vegan” (Oksanen et al. 2020) following (Lloyd-Price et al. 2019), including subject-level factors (constant across the samples of a subject) such as disease states, age, sex and race, and sample-level factors (recording status of the subjects in the week of sample collection) such as medication, antibiotics, immunosuppressant, chemotherapy, diarrhea, and bowel surgery (Supplementary Methods). The distances between the taxonomic composition of the samples were measured by Bray–Curtis dissimilarity. The distances between the microbial module activities of the subjects were measured by Euclidean distance.

The associations between the microbial modules and the clinical factors were identified. For each factor, modules that demonstrated significantly differential activities between distinct groups were detected from the factor matrix of subjects (H) using Wilcoxon rank-sum test with false discovery rate (FDR) ≤0.25. The modules associated with disease states (IBD-associated modules) were further analyzed. To investigate the roles of the taxa in the IBD-associated modules, IBD-associated microbial taxa were identified by (i) differential abundance (DA) analysis using Linear mixed-effects model (LMM) (Lloyd-Price et al. 2019), (ii) collecting microbe-disease associations from Human Microbe-Disease Association Database (HMDAD; Ma et al. 2017) and (iii) collecting IBD-associated taxa from other studies (Supplementary Methods). In addition, the top abundant pathways of each IBD-associated module were investigated for functional analysis. The abundance of each pathway contributed from the member taxa in all samples of the *“10-visit-set”* was summed up, and the top 20 abundant pathways were selected.

To study the relationships between the microbial taxa in the same module, the intramodule co-occurrence and functional similarity were investigated. The co-occurrence was measured by Dice Index (DI) and Spearman correlation, and the functional similarity was measured based on the contributed pathway abundance (Supplementary Methods). Specifically, we first calculated the co-occurrence for each taxa pair (i) individually by $\sum_{n}\left|C\left({\tilde{\chi }}_{nl.},{\tilde{\chi }}_{nl^{\prime }.}\right)\right|/N$ and (ii) across subjects by $\left|C\left(\mathrm{vec}({\tilde{\chi }}_{.l.}),\mathrm{vec}({\tilde{\chi }}_{.l^{\prime }.})\right)\right|$, where $C\left(x,x^{\prime }\right)$ was the DI or Spearman correlation between x and $x^{\prime }$, and vec(x) was the flattened vector of x. Then, the intramodule DI and Spearman correlation were computed by averaging the pairwise measures for each module. The intramodule functional similarity was computed by averaging the pairwise similarity of its members over all samples. In addition, we also estimated the pairwise correlations of microbial taxa using SparCC, a method proposed to estimate correlation values from compositional data to construct interaction networks of microbes (Friedman and Alm 2012). The sparcc function reimplemented by the SpiecEasi R package was used (Kurtz et al. 2015).

### 2.4 Classification of the subjects

To investigate the ability of the identified microbial modules to characterize the subjects, especially the IBD-associated modules, we evaluated the performance of the modules in stratifying the subjects into the IBD and control groups, as disease state characterization is of central importance. The module activities with respect to each subject (represented in the factor matrix H) were quantile normalized and used as features for classification. To compare the overall performance of different models and random runs, the top 100 modules that demonstrated the lowest *P*-value in associations with disease states were selected by Wilcoxon rank-sum test. The performance of using different numbers of top-ranking modules was evaluated. Random Forest (RF) with 2000 trees in the forest was adopted as the classifier. Other parameters of RF were tuned using the R package caret (Kuhn 2015). The classification performance was evaluated by the average area under the receiver operating characteristic curve (AUROC) of 10-fold cross-validation. In the analysis of IBD-associated modules, modules associated with disease states (Wilcoxon rank-sum test, FDR≤0.25) were used. For comparison, we evaluated the classification performance of the relative abundance of both differentially abundant taxa and all microbial taxa (Supplementary Methods). To thoroughly investigate the classification performance of relative abundance, we trained and tested RF models on the samples, the averaged profile, and randomly generated one-visit profiles of the *“10-visit-set”*, respectively. Area under the precision-recall curve (AUPR) was calculated as complementary for AUROC. Sensitivity, specificity, and precision were also evaluated by optimizing the cutoffs using Youden’s Index method (Youden 1950) implemented by the R package OptimalCutpoints (López-Ratón et al. 2014).

### 2.5 Validation of the microbial modules

The intramodule taxa relationships and the classification performance of the IBD-associated modules were further validated in the external datasets. The intramodule co-occurrence was evaluated in the validation sets, i.e. *“Val_Hall”*, *“Val_Lewis”*, and *“Val_HHS”* in the same way as in the discovery set discussed above. The classification performance of the IBD-associated modules was validated on *“Val_Hall”* which consisted of both IBD patients and healthy controls (Supplementary Fig. S1A–C). To estimate the module activities for the validation cohort, an incomplete third-order tensor $\chi^{\prime }$ was constructed from the samples in *“Val_Hall”*, and then factorized using the *visit-uncorrelated model*. The factor matrix of taxa extracted from the discovery set (M) indicating the identified microbial modules was used and fixed in the decomposition of $\chi^{\prime }$ to estimate the corresponding factor matrices of subjects ($H^{\prime }$) and visits ($T^{\prime }$) for the validation set (Supplementary Fig. S1D). In addition, to take full advantage of the knowledge derived from the discovery set and to make the validation results robust and reproducible, the average activities over the subjects and visits were calculated for each module and used as the initializations of the factor matrices $H^{\prime }$ and $T^{\prime }$, respectively. Suppose $C^{\prime }\subseteq \left\{1,\ldots ,C\right\}$ was the set of IBD-associated modules identified from the discovery set, the RF model with 2000 trees was trained on $H_{.C^{\prime }}$ and tested on ${H^{\prime }}_{.C^{\prime }}$ to evaluate the classification performance (AUROC and AUPR) of the IBD-associated modules on the validation set. As a comparison, the classification performance of relative abundance was also validated on *“Val_Hall”*, where RF was trained on all samples in the *“10-visit-set”*, and tested on all samples, the averaged profile and the one-visit profiles (evaluated by the average AUROC/AUPR over ten randomly generated one-visit profiles) of the validation set, respectively. The corresponding sensitivity, specificity, and precision were also calculated based on Youden’s Index for the validation results.

## 3 Results

### 3.1 Decomposition of longitudinal gut microbiota to identify microbial modules

The workflow of the study is illustrated in Fig. 1. The human longitudinal gut microbiota data of 97 subjects, including 72 IBD patients (47 CD and 25 UC) and 25 healthy controls, was collected from integrative Human Microbiome Project et al. (2019) and used as the discovery set (Fig. 1A and B). Two sets were generated from the discovery set, of which the *“10-visit-set”* consisted of 10 samples from each subject with roughly equal sampling intervals, whereas the *“24-visit-set”* involved all samples of the subjects (Fig. 1C–E). The *“10-visit-set”* included about 66% of the samples in the *“24-visit-set”*.

To identify the underlying microbial modules, we represented the samples in the *“10-visit-set”* as a complete third-order tensor $\tilde{\chi }$ and factorized the tensor into three factor matrices in terms of the subjects (H), taxa (M), and visits (T) (Fig. 1F) using the *visit-correlated model* which accounted for the correlations between the samples collected from the same subject (Fig. 2A). Thereby, the taxonomic composition of the gut microbiota in a subject over time was factorized into the contributions of multiple microbial modules. The factor matrices were then used for microbial module identification, module analysis, and subject stratification. As a comparison, a *visit-uncorrelated model* was implemented (Supplementary Fig. S2) and used to factorize $\tilde{\chi }$ as well as the incomplete third-order tensor $\hat{\chi }$ constructed from *the“24-visit-set”*. The factorization results were referred to as *“10-visit-correlated”*, *“10-visit”*, and *“24-visit”*, respectively (Table 1).

We compared the performance of the top-ranking microbial modules identified using different models and data in stratifying the subjects into IBD and healthy controls to demonstrate their ability in gut microbiota characterization. The AUROC increases when more modules are used to stratify the subjects (Fig. 2B). For the *visit-uncorrelated model* not accounting for the correlation among samples from the same subjects, the results of the *“24-visit-set”* outperform that of the *“10-visit-set”*, indicating that involving mores samples improves the classification performance, probably due to the more comprehensive information of the subjects (Fig. 2C). The performance is further improved by the results of the *visit-correlated model* applied to the *“10-visit-set”* although fewer samples are included compared with the *“24-visit-set”*, revealing the benefit of modeling the correlations of repeated measures.

In order to determine the final microbial modules for downstream analysis, we compared the results of different runs of *“10-visit- correlated”*. The result of “run_10” was selected on account of (i) the best overall classification performance and (ii) the lowest reconstruction error (RMSE), i.e. the differences between the original abundance tensor and the results of factorization (Fig. 2D). A total of 287 modules were identified from the results of “run_10” for further analysis. On average, there are 15.03 taxa included in each module.

### 3.2 Interindividual variation of the microbial modules

**Figure 3:**
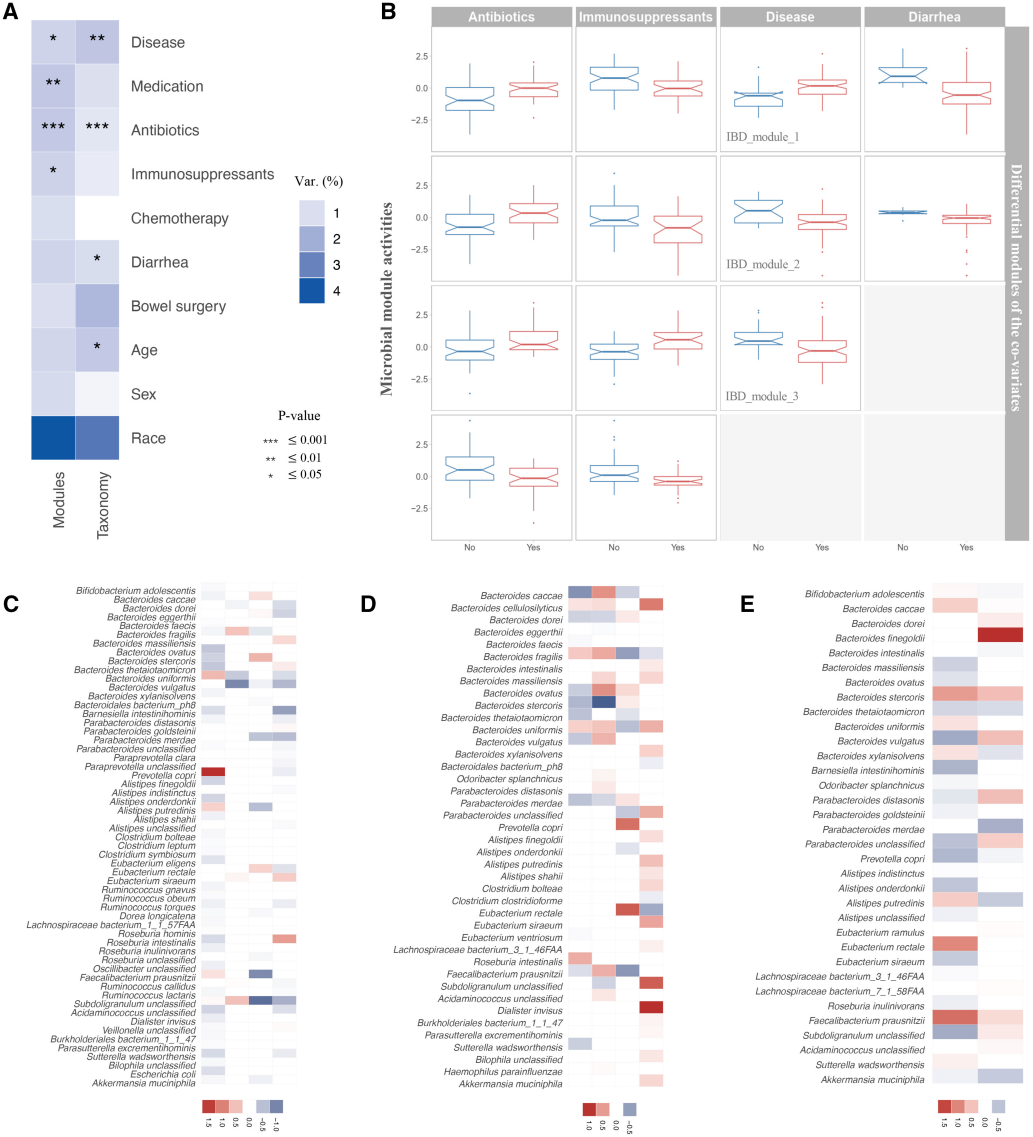
Relationships between the microbial modules and clinical factors. (A) The variation in terms of the microbial module activities and the taxonomic composition explained by several clinical factors, quantified by PERMANOVA. The color represents the proportion of variance explained by each factor. (B) Associated modules of antibiotics, immunosuppressants, disease states, and diarrhea, respectively (Wilcoxon rank-sum test, FDR≤0.25). Each column lists the associated modules of one factor, and each panel demonstrates the activities of a module in distinct groups (“Yes” and “No”) of the associated factor. Member taxa of the associated modules of antibiotics (C), immunosuppressants (D), and diarrhea (E), respectively. Each column represents a module. The color demonstrates the activities of the taxa in the modules.

By factorizing the composition of gut microbiota into the contribution of microbial modules, we characterized gut microbiota by microbial modules instead of individual microbial taxa. To explore the interindividual dissimilarity, we quantified the variation of microbial module activities explained by several clinical factors, including disease states, medication, antibiotics, immunosuppressant, chemotherapy, diarrhea, bowel surgery, age, sex, and race, using PERMANOVA, and compared with the intersample variation of taxonomic composition. The subjects' clinical information in *the“10-visit-set”* is summarized in Supplementary Table S1. In general, each factor only explains a small proportion of the interindividual variation of the microbial module activities, consistent with the situation of taxonomic compositions (Fig. 3A). Race explains the highest variation of both the module activities (4.01%) and taxonomic compositions (3.28%) among the factors, indicating the ethnic differences in gut microbiota related to lifestyle, diet, etc. (Deschasaux et al. 2018; Amato et al. 2021) Medication, antibiotics and immunosuppressants (1.23%–1.43%), as well as disease states (1.22%), contribute more to the diversity of the module activities with higher significance compared with the other factors (≤1.19%), implying their greater influences on gut microbiota.

Some sample-level clinical factors, e.g. chemotherapy and bowel surgery, demonstrate more significant differences in the contributions to taxonomic composition and module activities, possibly suggesting distinct perturbation effects to the hosts. For each sample, the taxonomic composition and clinical information reflect the temporary changes and status of the subject in the week of sample collection. As the module activities of a subject are summarized from the longitudinal samples, they provide an overall characterization of the subject in the whole sampling period, and the overall states of the clinical factors were used for the variance analysis (Supplementary Methods). Therefore, the contributions of the clinical factors to taxonomic composition and module activities imply their influences on the short- and long-term changes of gut microbiota, respectively. For example, bowel surgery explains a larger proportion of taxonomic variation (1.79%) among the factors, but less proportion of module activity variation (1.0%). One possible reason is that the gut microbiota is dramatically changed in the bowel preparation and early postoperative period, and then recovers to the baseline after a few weeks with less long-lasting changes to the hosts (Bachmann et al. 2017; Nalluri-Butz et al. 2022). On the contrary, chemotherapy explains a low proportion of taxonomic variation (0.33%) and a higher proportion of module activity variation (1.01%). Several studies have reported that gut microbiota would be altered after chemotherapy treatments and recover to new communities which are highly dissimilar to the baseline, indicating the long-term effects of chemotherapy on gut microbiota (Chua et al. 2020; Rashidi et al. 2022).

### 3.3 Association analysis of the microbial modules

We first investigated the associations between the microbial modules and the above-mentioned clinical factors. The modules demonstrating differential activities (Wilcoxon rank-sum test, FDR≤0.25) in distinct groups of each factor were identified. We linked four modules with each of antibiotics and immunosuppressants use, three modules with disease states and two modules with diarrhea (Fig. 3B). Different modules were detected associated with the same factor, possibly due to the different effects of the factor on the modules and the interindividual heterogeneity. The activities of the members in each module are illustrated in Fig. 3C–E, except the IBD-associated modules, which are discussed in detail later. The modules suggest groups of microbial taxa altered together by the corresponding factor, providing candidates for mechanism study and therapeutical interventions. For instance, as the most active species in the antibiotics-associated modules, *Prevotella copri* has been reported changed abundance under antibiotic treatment (Zhang et al. 2019; Péan et al. 2020), and *Bacteroides vulgatus* has been found sensitive to antibiotic exposure (Cuisiniere et al. 2021). *Dialister invisus*, *Bacteroides stercoris*, and *Eubacterium rectale*, which contribute a lot to the immunosuppressant-associated modules, have been previously linked to autoimmune and inflammatory conditions (Culina et al. 2018; van den Munckhof et al. 2018; Wang et al. 2018; 2021; Leonard et al. 2021; Yeoh et al. 2021). For the microbial taxa included in the diarrhea-associated modules, *Bacteroides finegoldii* has been reported crucial to intestinal barrier damage prevention (Leng et al. 2021). The relative abundance of genera *Eubacterium*, *Faecalibacterium*, *Bifidobacterium*, and *Bacteroides* in the diarrheal fecal specimens significantly differs from the control group (Lee et al. 2019). The relative abundance of these active taxa in distinct groups of the corresponding clinical factors is shown in Supplementary Fig. S3. In addition, the activities of these microbial modules in the subtype groups of IBD (CD and UC) and the control group are illustrated in Supplementary Fig. S4A–C. The differences in the module activities are mainly dominated by the states of the corresponding factors rather than the disease states. Each diagnostic class demonstrates increased/decreased activities in the case group compared with the control group of each clinical factor. The results further support the principal effects of the clinical factors on the microbial modules.

**Figure 4:**
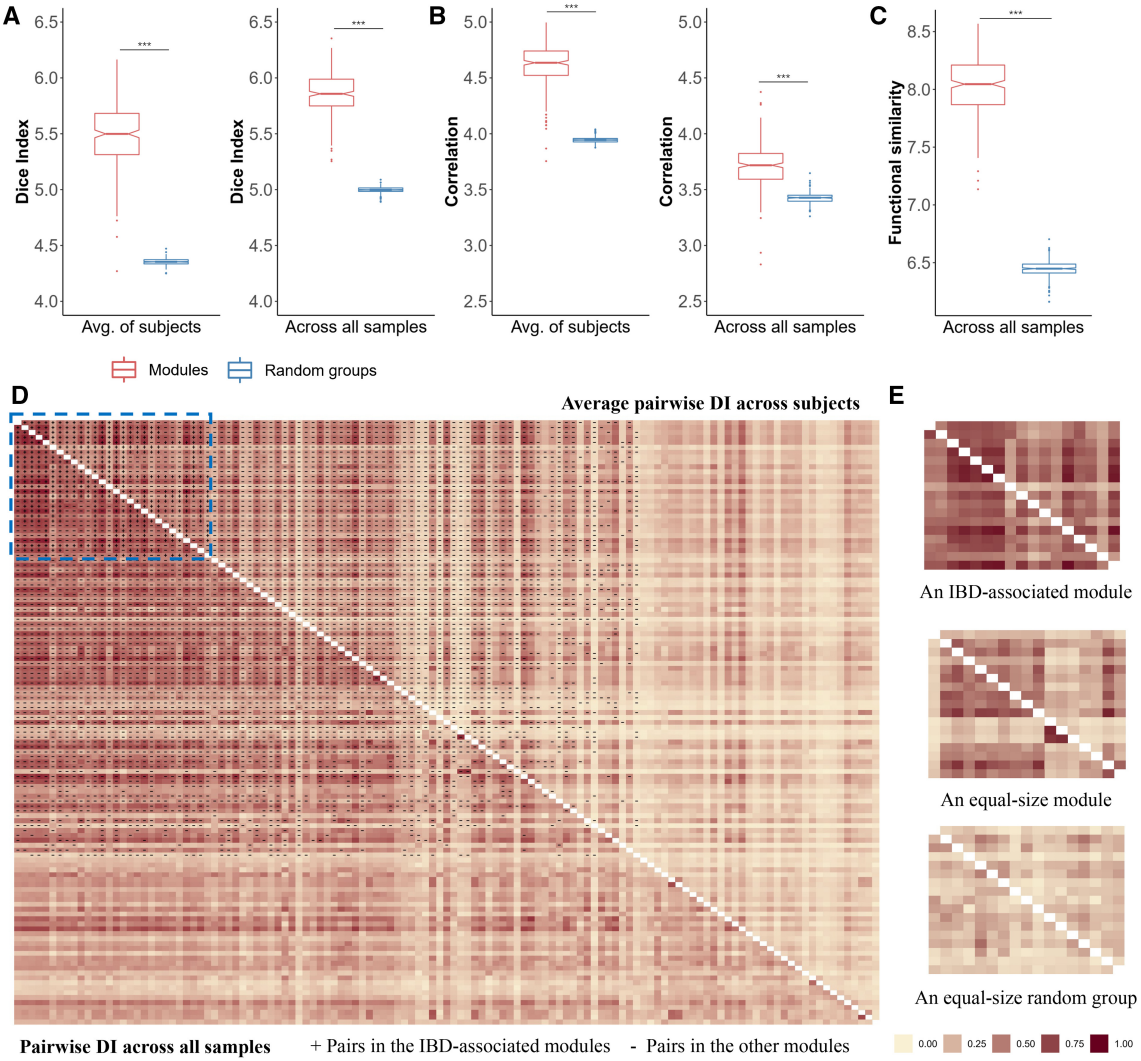
Intramodule associations of the member taxa. The intramodule co-occurrence of taxa measured by DI (A) and absolute Spearman correlation (B) calculated individually (average of subjects) and across all samples, compared with that of equal-size random groups formed by the taxa pairs not included in any module. (C) The intragroup functional similarity of the modules calculated across all samples. ***, *P*-value ≤ .001. The values are multiplied by a scaling factor of 100 and then log-transformed. (D) Pairwise DI of all taxa calculated individually (upper triangle) and across all samples (lower triangle). Taxa pairs in the IBD-associated modules (+) are distributed in the blue rectangle. (E) Examples of the pairwise DI of an IBD-associated module, an equal-size module, and an equal-size random group, respectively.

Since the modules represent groups of related taxa with similar underlying patterns affected by the same latent factor, we examined the intramodule associations of the taxa. Compared with the random groups formed by the taxa pairs not included in any module, the identified microbial modules demonstrate significantly higher intragroup DI, relative abundance correlation over time (measured by the absolute Spearman correlation), and similarity of pathway contributions (Fig. 4A–C), indicating that the taxa in the same module are highly correlated and participate in the same pathways. In particular, the modules include most taxa pairs with relatively high co-occurrence, especially the IBD-associated modules (Fig. 4D and E and Supplementary Fig. S5). The pairwise correlation coefficients of microbial taxa estimated by SparCC demonstrate consistent results (Supplementary Fig. S6), confirming the close relations of the taxa in the same module.

### 3.4 Analysis of IBD-associated microbial modules

**Figure 5:**
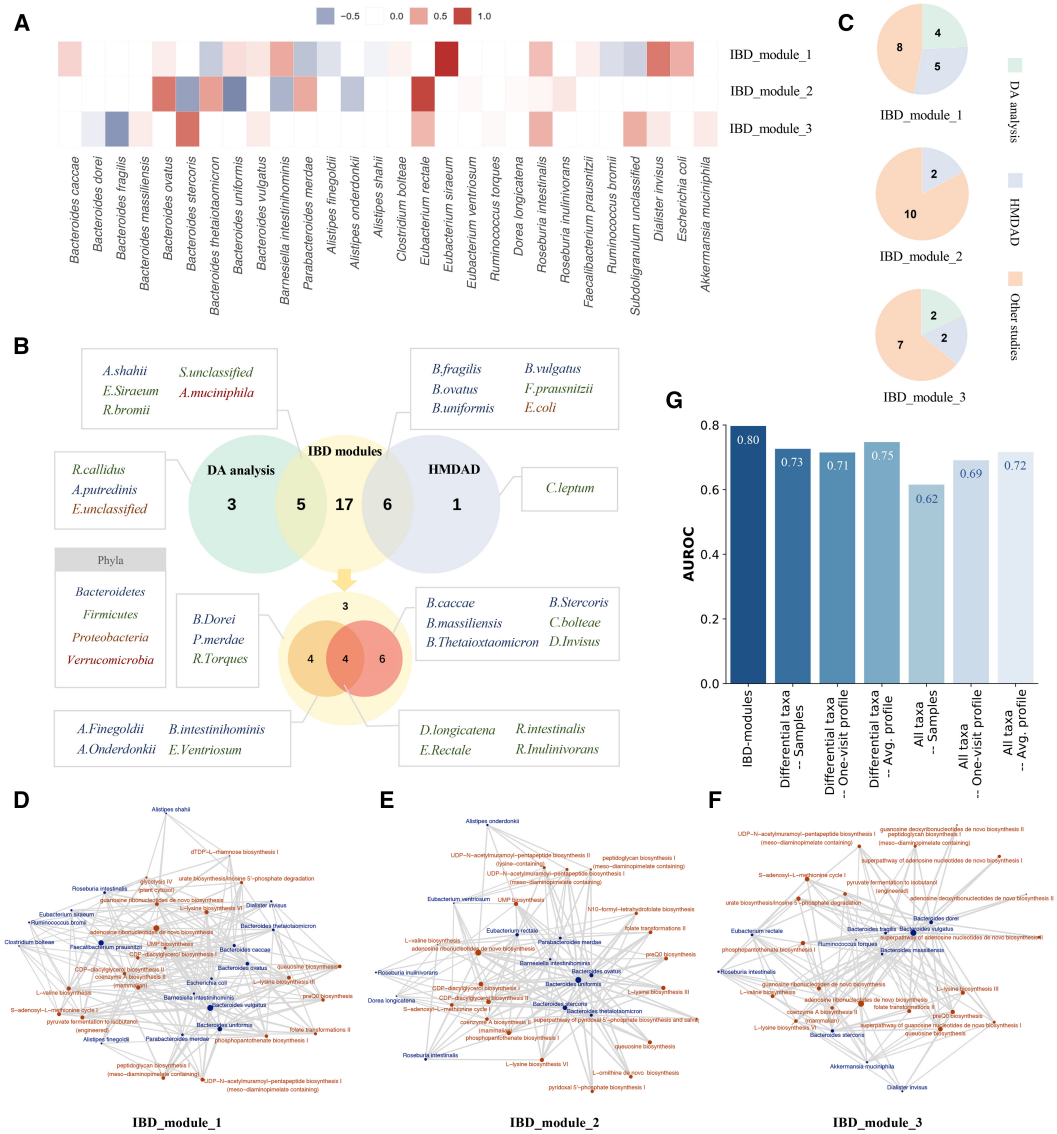
The IBD-associated modules. (A) The activities of the member taxa in each IBD-associated module. (B) The association support from different sources. Top, the venn plot comparing the taxa in the IBD-associated modules, the IBD-associated taxa detected by DA analysis and the taxa reported associated with IBD in HMDAD. Bottom, the venn plot demonstrating the supports from another two studies. The color of the species indicates the phyla they belong to. (C) The pie chart of the number of taxa supported by each source in each IBD-associated module. (D–F) The contributions of the member taxa to the top pathways in each IBD-associated module. Blue circles, microbial taxa. Brown circles, the top 20 abundant pathways. Size of the circles, the abundance of the pathways (for pathway circles), or the contribution of the taxa to the pathways (for the taxon circles). Lines, contributions of the taxa to the pathways. (G) The classification performance (AUROC) of the IBD-associated modules and taxa relative abundance in the discovery set.

We identified three microbial modules demonstrating differential activities in the IBD and control groups (Wilcoxon rank-sum test, FDR≤0.25), i.e. IBD_module_1, IBD_module_2, and IBD_module_3 (Fig. 3B), and two of them (IBD_module_1 and IBD_module_3) also distinguish the control group from each subtype of IBD (Kruskal–Wallis test, FDR≤0.25; Supplementary Fig. S4D). The activities of the members in the modules are illustrated in Fig. 5A. Several species demonstrate higher importance in the IBD-associated modules, such as *Eubacterium siraeum* in IBD_module_1, *E. rectale*, and *Bacteroides uniformis* in IBD_module_2, and *Bacteroides fragilis* and *B. stercoris* in IBD_module_3. The 3 modules are composed of 28 taxa, mainly belonging to the phyla of *Bacteroidetes* and *Firmicutes*. All of the taxa are associated with IBD based on DA analysis and previous studies (Fig. 5B and C and Supplementary Tables S2–S6). Specifically, five taxa are detected by DA analysis of the discovery set using LMM, and the associations of another six are indicated in HMDAD. Among the remaining 17 taxa, 14 are reported as differentially abundant taxa in two IBD studies (Franzosa et al. 2019; Ma et al. 2021). For the other three taxa, literature validations are also found by searching PubMed (Vidal et al. 2015; Takahashi et al. 2016; Nomura et al. 2021). The log-transformed relative abundance of the taxa in the *“10-visit-set”* is illustrated in Supplementary Fig. S7.

The top 20 abundant pathways of each IBD-associated module as well as the contributions of the member taxa to the pathways are demonstrated in Fig. 5D–F and Supplementary Fig. S8. The most abundant pathway in all three modules is *adenosine ribonucleotides de novo biosynthesis*, which is involved in many basic biochemical processes (Schirmer et al. 2018). Some other abundant pathways include *coenzyme A biosynthesis II*, *phosphopantothenate biosynthesis I*, *CDP-diacylglycerol biosynthesis*, and *dTDP-L-rhamnose biosynthesis I*, which play crucial roles in therapeutic interventions and host immune response. Coenzyme A (CoA) is a cofactor of ubiquitous occurrence in many living organisms involved in a large number of enzymatic reactions (Rubio et al. 2006). It is recognized as a target for antibacterial drug discovery (Leonardi et al. 2005). 4'-phosphopantothenate, the product of *phosphopantothenate biosynthesis I*, is the universal precursor for synthesizing the 4'-phosphopantetheine moiety of CoA. Pantothenate in the pathway could only be synthesized by plants and microorganisms (Begley et al. 2001), and the enzymes of this pathway are therefore considered to be antimicrobial drug targets. The metabolism of phospholipids, which is synthesized and remodeled through pathways including *CDP-diacylglycerol*, represents a highly controlled cellular signaling network that is essential for mounting an effective innate immune response (O’Donnell et al. 2019). In *dTDP-L-rhamnose biosynthesis I*, β-L-rhamnopyranose is produced as a building block of the glycan component of the O-antigens, a major target of the immune system (Schirmer et al. 2018).

The classification performance of the three IBD-associated modules was compared with that of the relative abundance of microbial taxa (Fig. 5G). For relative abundance, we evaluated the performance of the differentially abundant taxa (8 species) detected by LMM and all taxa (123 species) in stratifying (i) all samples, (ii) the averaged profile, and (iii) the randomly generated one-visit profiles, respectively (Supplementary Methods). The performance of all taxa (average AUROC = 0.68) is inferior to that of the differentially abundant taxa (average AUROC = 0.73), possibly due to the overfitting issue. The IBD-associated modules (AUROC = 0.80) outperform the relative abundance (AUROC ≤ 0.75) in the discovery set, indicating that they better distinguish between the gut microbiota of the IBD and control groups and are likely better depictions of gut microbiota. The evaluated AUPR demonstrates consistent results with AUROC (Supplementary Fig. S9A). The results of other metrics, including sensitivity, specificity, and precision, are summarized in Supplementary Table S7.

### 3.5 Validation of microbial modules in external cohorts

**Figure 6:**
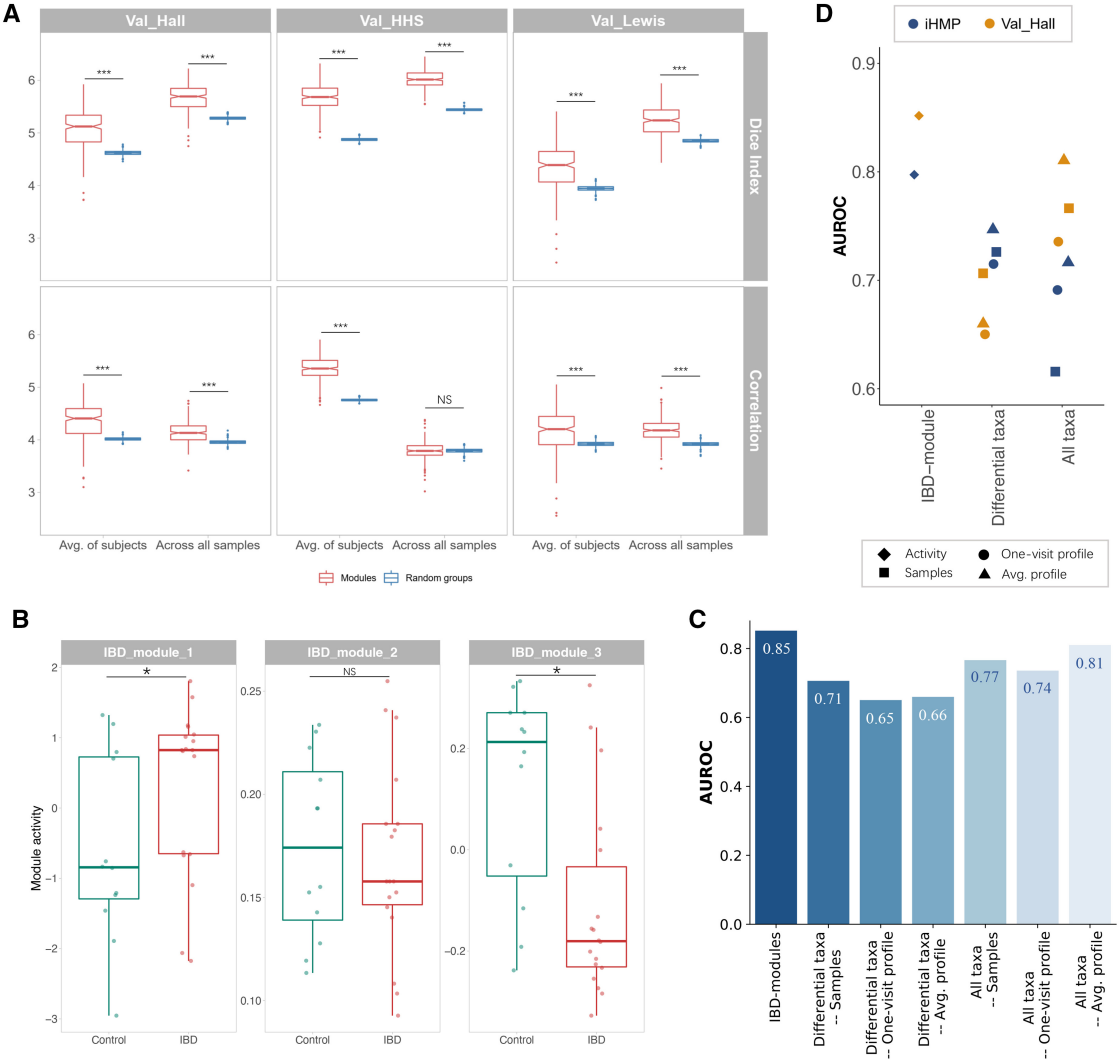
Validation of the microbial modules in the external cohorts. (A) The intramodule co-occurrence in the validation cohorts measured by DI and absolute Spearman correlation, compared with that of equal-size random groups formed by the taxa pairs not included in any module. ***, *P*-value ≤ .001. NS, not significant. (B) The activities of the IBD-associated modules in the control and IBD groups of the validation set *“Val_Hall”*. *, FDR ≤ 0.25. (C) The classification performance (AUROC) of the IBD-associated modules and the taxa relative abundance in *“Val_Hall”*. (D) Comparison of the classification performance (AUROC) in the discovery set (iHMP) and the validation set *“Val_Hall”*.

To validate the microbial modules identified from the discovery set, we estimated the intramodule co-occurrence of taxa in three validation sets (Table 1) and evaluated the classification performance of the IBD-associated modules in *“Val_Hall”*. As in the discovery cohort, the modules also demonstrate significantly higher intragroup DI and Spearman correlation in the validation cohorts compared with the random groups, which confirms the close relationships between the taxa in the same module (Fig. 6A and Supplementary Fig. S10). The relative abundance of taxa in the IBD-associated modules in the validation set *“Val_Hall”* are illustrated in Supplementary Fig. S11.

Among the three IBD-associated modules identified from the discovery set, two of them (IBD_module_1 and IBD_module_3) also demonstrate significantly differential activities (FDR ≤0.25) between the IBD and control groups in the validation cohort, and retain the same patterns as in the discovery set, i.e. higher activities in the IBD group (IBD_module_1) and control group (IBD_module_3), respectively (Figs 3B and 6B). We compared the classification performance of the three IBD-associated modules to that of the microbial taxa relative abundance in the validation set, where RF models were trained on the discovery set and tested on *“Val_Hall”*. Contrary to the results in the discovery set, the relative abundance of all taxa (average AUROC = 0.77) performs better than that of the differentially abundant taxa identified from the discovery set (average AUROC = 0.67), indicating that the differentially abundant taxa are not the most discriminative taxa in the validation set anymore (Fig. 6C and D). In fact, among the eight differentially abundant taxa, only one (*Alistipes putredinis*) demonstrated DA between the two groups in the validation cohort, in line with the previous discovery that inconsistent associations are reported in different cohorts and studies (Duvallet et al. 2017; Wirbel et al. 2019; Nearing et al. 2022). The IBD-associated modules (AUROC = 0.85) are superior to the relative abundance (AUROC ≤ 0.81), revealing their efficacy in other cohorts and the great promise of the proposed method in identifying common microbial modules from human gut microbiota. The results also suggest that it might be more reliable to investigate the gut microbiota using microbial modules instead of individual microbial taxa. The AUPR also demonstrates consistent results in the validation cohort (Supplementary Fig. S9B). The sensitivity, specificity, and precision determined by Youden’s Index are summarized in Supplementary Table S8.

## 4 Discussion

In this study, we proposed a tensor factorization-based method to identify the underlying microbial modules from longitudinal gut microbiota data and applied it to IBD. The taxonomic composition was factorized into the contributions of multiple microbial modules where spike-and-slab prior was adopted to group taxa with similar abundance patterns. The correlations of repeated measures in longitudinal data were also accounted for. The taxa members in the same module demonstrate higher co-occurrence and functional similarity, revealing their close relationships and potential microbe–microbe interactions. The modules with differential activities between distinct groups in terms of several clinical factors, including disease states, antibiotics, immunosuppressants, and diarrhea, were identified, respectively. All of the taxa in the IBD-associated modules are linked to IBD in previous studies or demonstrate DA between the IBD and control groups. The performance of the IBD-associated modules in stratifying the subjects is superior to taxa relative abundance in both the discovery set and the validation set, indicating that the IBD-associated modules well capture the alterations in disease conditions and are more robust across cohorts. The results reveal the benefit of microbial modules and their promise in uncovering the underlying structures of gut microbiota and deciphering associations between gut microbiota and clinical factors.

The identified association between a microbial module and a clinical factor suggests potential influences of the factor on the whole taxa group through direct regulation and microbe–microbe interactions. For some modules, there are a few active taxa with remarkably high absolute activities among the others, which are probably leading taxa of the modules, e.g. *P. copri* in an antibiotics-associated module and *B. finegoldii* in a diarrhea-associated module. Evidence of the associations between the active taxa and the corresponding factors has been reported in previous studies (Zhang et al. 2019; Péan et al. 2020; Cuisiniere et al. 2021). As the microbial modules represent the underlying patterns in terms of individual latent factors, whereas the relative abundance of taxa demonstrates the overall outcome of the factors, the abundance patterns of the member taxa might differ from the activity pattern of the corresponding module. Even for the most active member taxa in a module, their underlying patterns might also be masked and obscured in the relative abundance due to the effects of other factors. The relative abundance of individual taxa might not be a robust way to study gut microbiota alterations and associations with clinical factors because of the compositional format, intricate effects of various latent factors and ecological interactions. It is further validated in this study by the inconsistent differential taxa of disease states between the discovery and the validation cohorts as well as the inferior classification performance compared with microbial modules.

Longitudinal data of gut microbiota explored in this study facilitate capturing the co-occurrence of the microbial taxa, including not only the co-existence but also the co-varying relationships. The taxa in the same module tend to co-vary in the relative abundance caused by the influences of the underlying factor, which are signified by the higher intragroup pairwise absolute Spearman correlation of the modules, even though the patterns are diluted in observations by other factors. However, the result of the microbial modules might be affected by the alignment of the samples from different subjects in the tensor. It is difficult to determine a baseline or starting time point for all subjects to align the samples. Some temporal alignment methods for longitudinal microbiota data have been proposed to map the time series data from different subjects to the same time scale to unify the distinct rates of change (Lugo-Martinez et al. 2019), but there are some limitations in aligning the samples. Remodeling and scaling of the time series data during the alignment might involve additional noises. Moreover, some studies suggest that the change rate of microbiota itself might serve as a clinical feature, which is an essential aspect of gut microbiota characterization (Gajer et al. 2012; Gilbert et al. 2018) but possibly eliminated by the alignment methods. Therefore, we did not perform time alignment before microbial module identification. The results of this study demonstrate that the proposed method is able to identify representative and robust microbial modules without time alignment.

In the proposed method, the longitudinal data was modeled as a third-order tensor where the samples from the same subject were placed in the same slice, and the correlations between them were accounted for, which improved the results compared with the *visit-uncorrelated model*. In addition, we also implemented the 2D version of the proposed method where the samples in the *“10-visit-set”* were arranged in a matrix modeling neither the relationships between the samples and the subjects nor the correlations between the repeated measures. The relative abundance matrix was factorized using the model illustrated in Supplementary Fig. S12 to identify microbial modules. In general, the microbial modules identified using the proposed method demonstrate higher intragroup co-occurrence than the 2D version (Supplementary Fig. S13). Furthermore, no IBD-associated module was detected from the results of the 2D version by LMM with FDR≤0.25. The results additionally validate the advantages of accounting for the relationships between the samples in the proposed method.

The microbial modules provide insights into underlying factors that collectively determine human gut microbiota composition, which deserves further investigation. Our results demonstrate the benefit and great promise of group analysis of gut microbial taxa toward conventional association study of individual taxa, emphasizing the significance of ecological effects in understanding the relationships of gut microbiota with human health. The study has demonstrated the efficacy of the method in association analysis of longitudinal gut microbiota data in inflammatory bowel disease, suggesting its potential in other disease scenarios. The proposed method is also applicable to other types of omics data as it does not involve any specific prior knowledge and designs of metagenome (Cheng and Leung 2018; Cheng et al. 2018; Liu et al. 2021).

## Supplementary Material

btad213_Supplementary_Data

## Data Availability

The longitudinal gut microbiota data used as the discovery set in this study is available at https://www.ibdmdb.org. The validation datasets are available at BioProject with accession number P RJNA385949 (“*Val_Hall*”), NCBI SRA with accession number SRP057027 (“*Val_Lewis*”) and https://www.hmpdacc.org/hmsmcp2/ (“*Val_HHS*”), respectively.
