# Supplementary material for: Deciphering associations between gut microbiota and clinical factors using microbial modules
Source: Bioinformatics. 2023 Apr 21;39(5):btad213. doi: 10.1093/bioinformatics/btad213 (PMC10191612; doi:10.1093/bioinformatics/btad213)
Supplement: btad213_Supplementary_Data [file btad213_supplementary_data.pdf]

Supplementary Information for
"Deciphering associations between gut microbiota and clinical factors using microbial modules"

Ran Wang, Xubin Zheng, Fangda Song, Man Hon Wong,
Kwong Sak Leung, Lixin Cheng

Contents

Supplementary Figures 2
Supplementary Tables 15
Supplementary Methods 19
  Identification of gut microbial modules 19
  Relationships between the microbial taxa in the modules 23
  Permutational multivariate analysis of variance (PERMANOVA) 23
  IBD-associated microbial taxa 24
  Classification of the subjects using relative abundance 24
References 25

Supplementary Figures

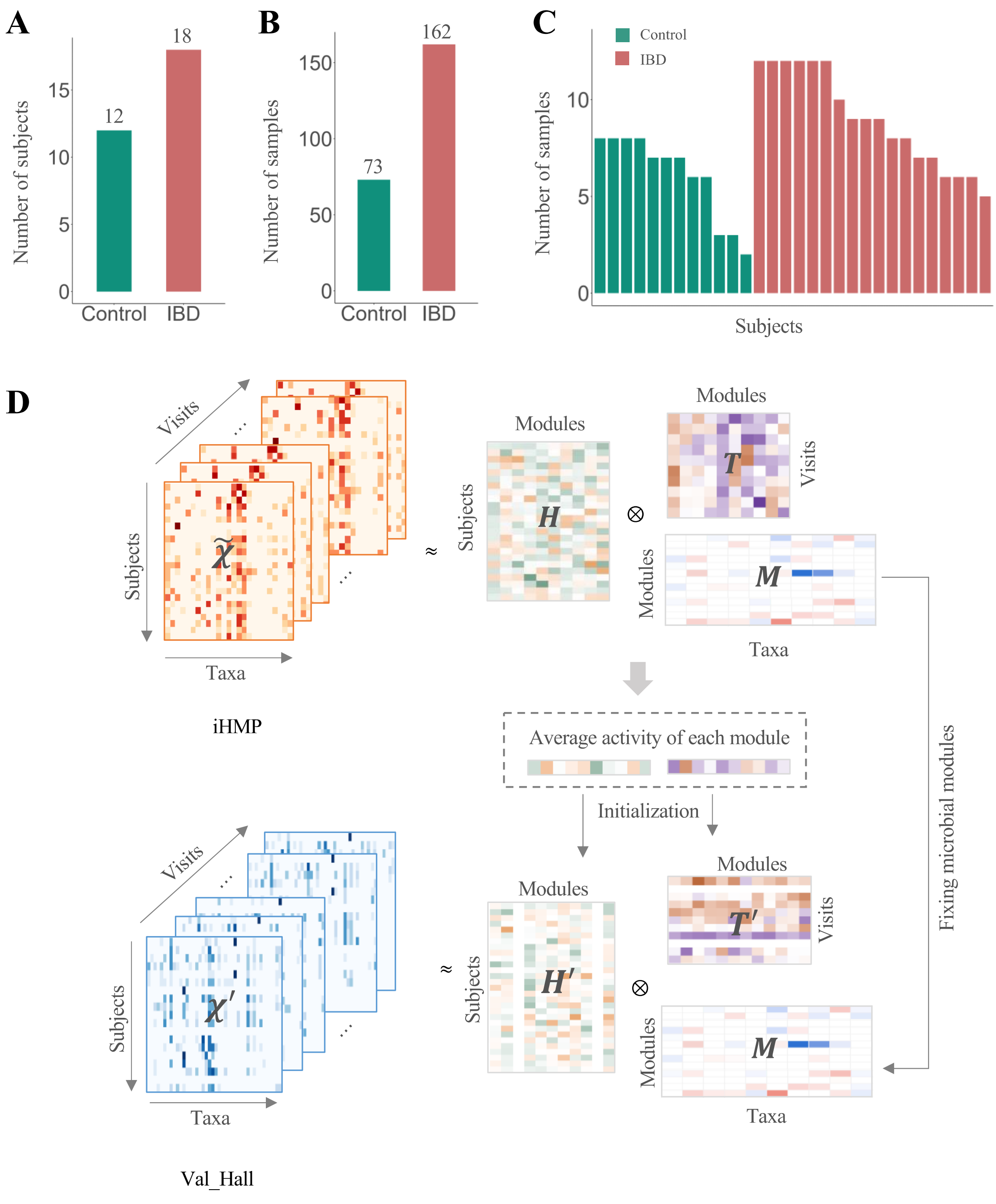

Supplementary Fig. S1. Validation of the microbial modules in the *"Val_Hall"* set. The number of subjects (**A**) and samples (**B**) in the control and IBD groups of *"Val_Hall"*. **C.** The number of samples collected from each subject. **D.** Illustration of estimating the activities of the microbial modules in the validation cohort. The factor matrix of microbial taxa M identified from the discovery set (iHMP) is fixed in the factorization of the validation set. The average activities of each module across the subjects and visits are used as the initializations in the factorization of the validation set.

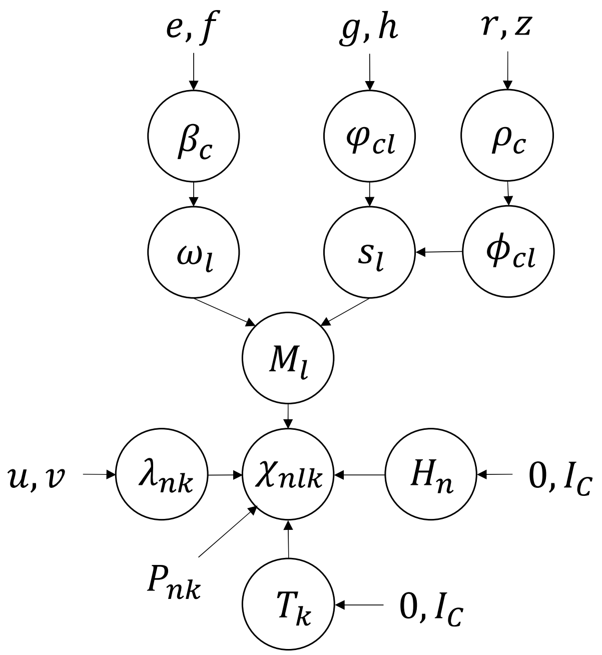

Supplementary Fig. S2. The Bayesian tensor factorization model of *visit-uncorrelated*.

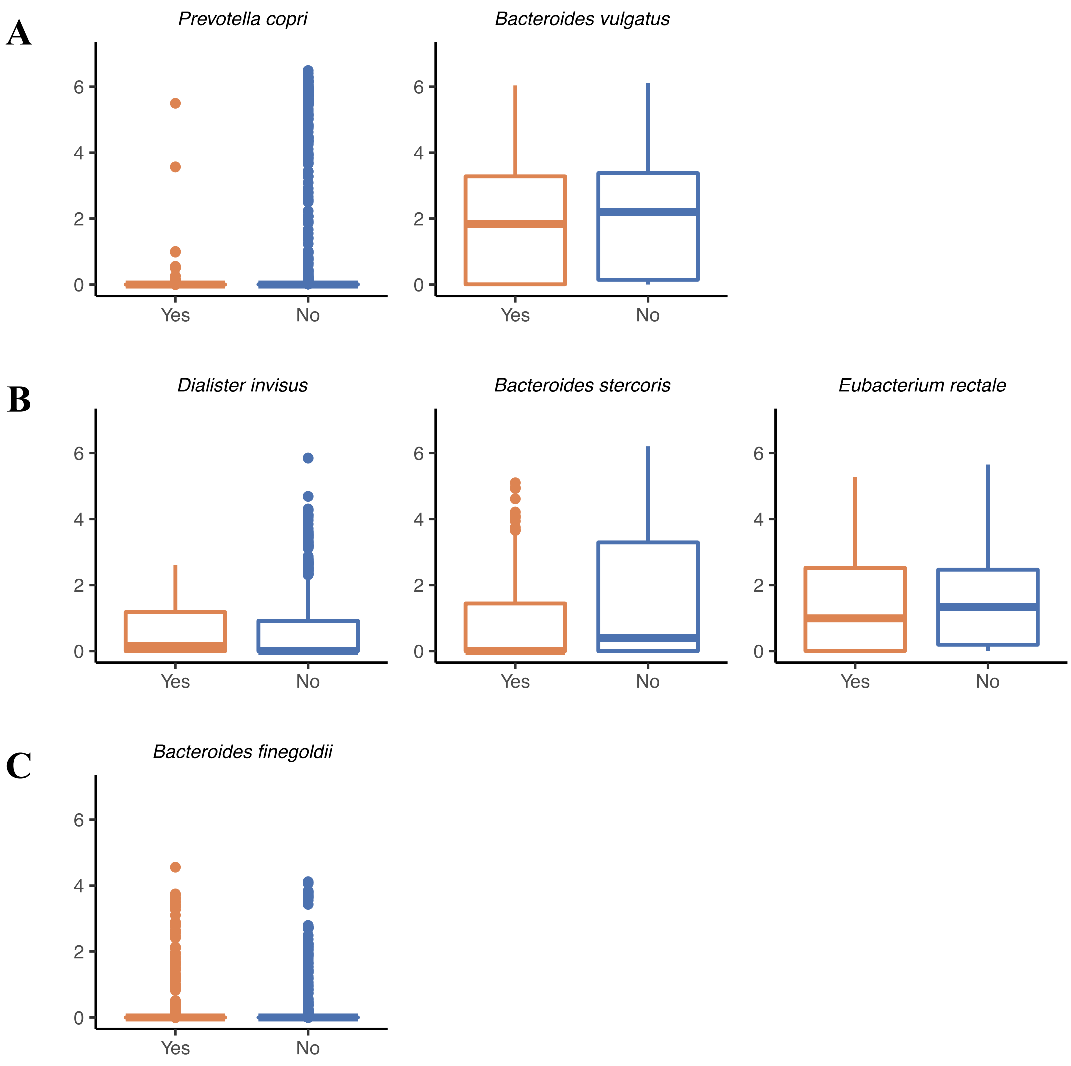

Supplementary Fig. S3. Relative abundance (log-transformed) of the most active taxa in the modules associated with antibiotics (A), immunosuppressants (B) and diarrhea (C). "Yes" and "No" indicate the distinct groups of clinical factors.

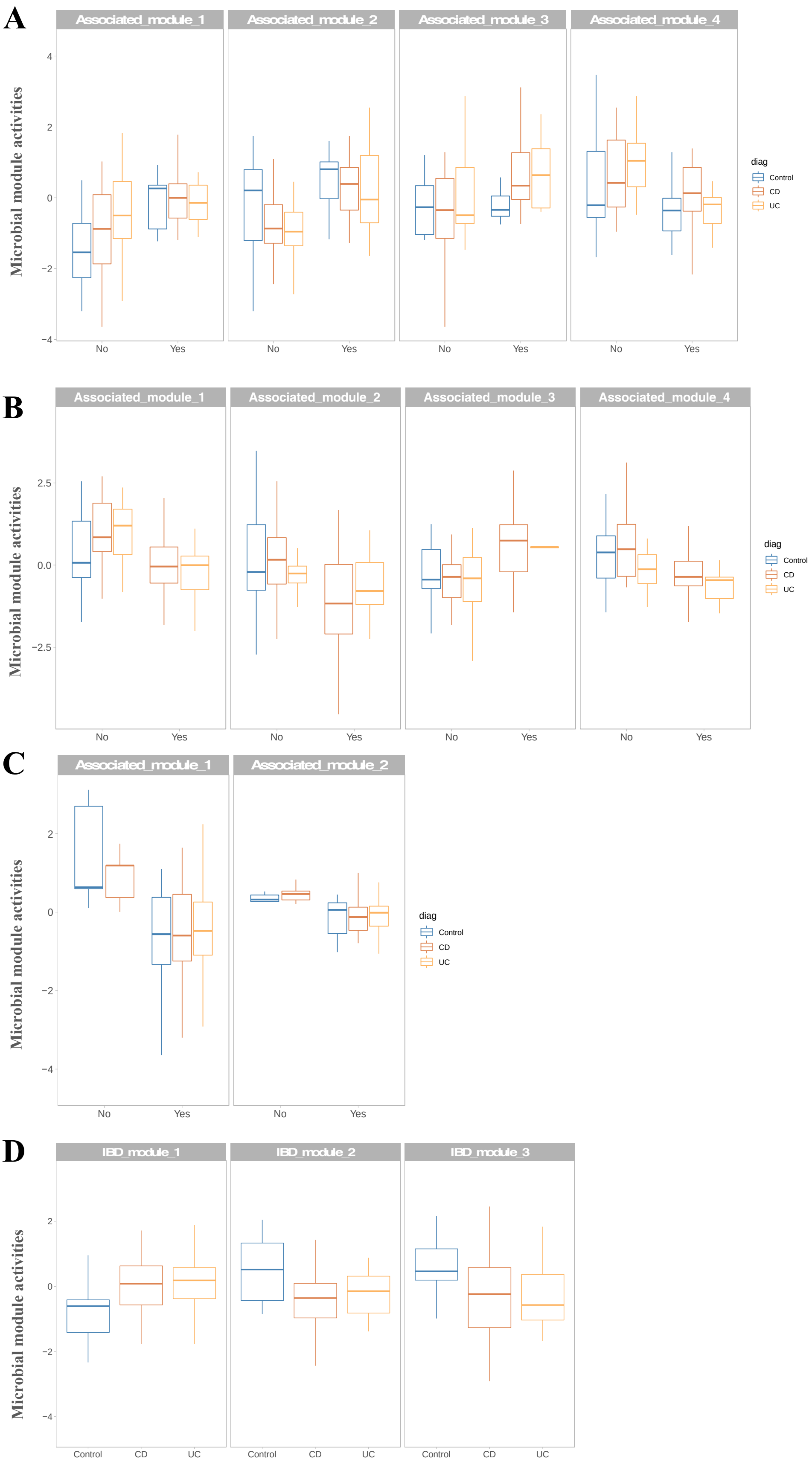

Supplementary Fig. S4. Activities of the differential modules in terms of antibiotics (A), immunosuppressants (B), diarrhea (C) and disease states (D) in the control, CD and UC groups. “Yes” and “No” indicate the different states of each clinical factor.

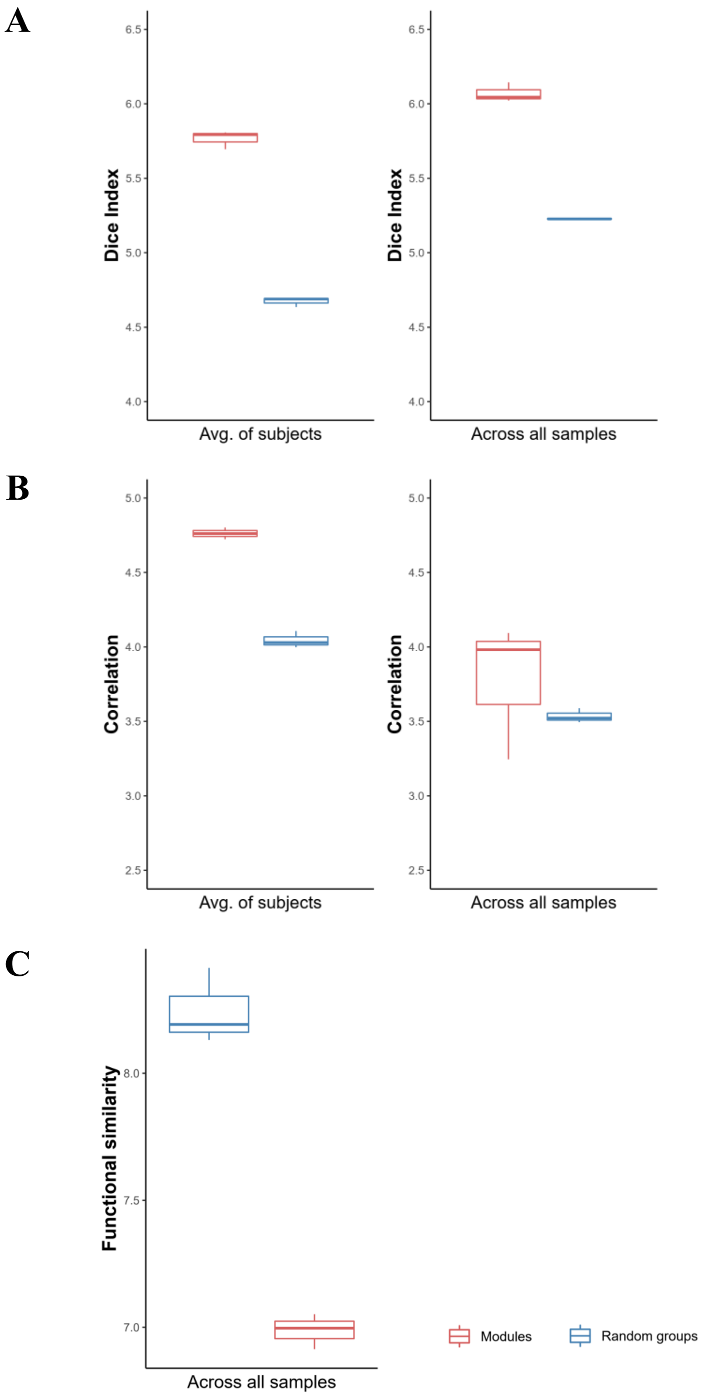

Supplementary Fig. S5. The intra-module Dice Index (A), absolute Spearman correlation (B) and functional similarity (C) of the IBD-associated modules compared to the equal-size random groups.

A

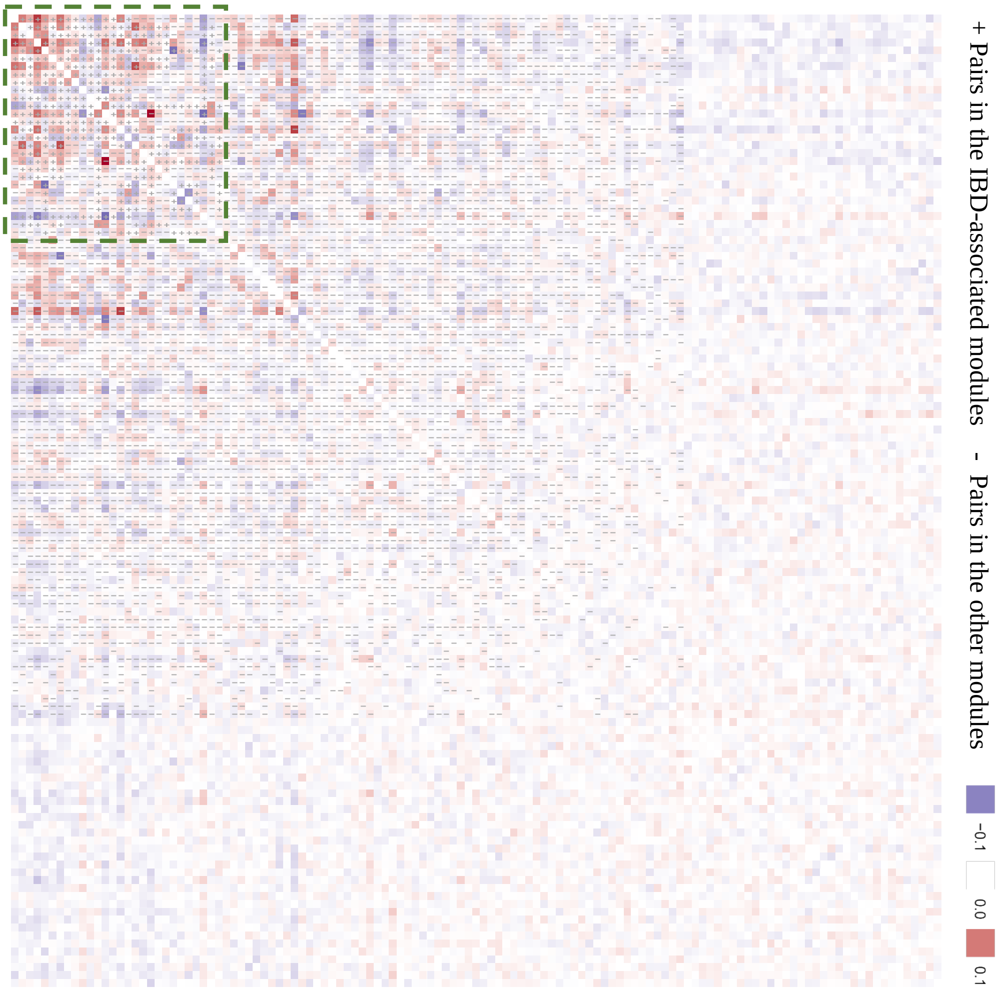

B

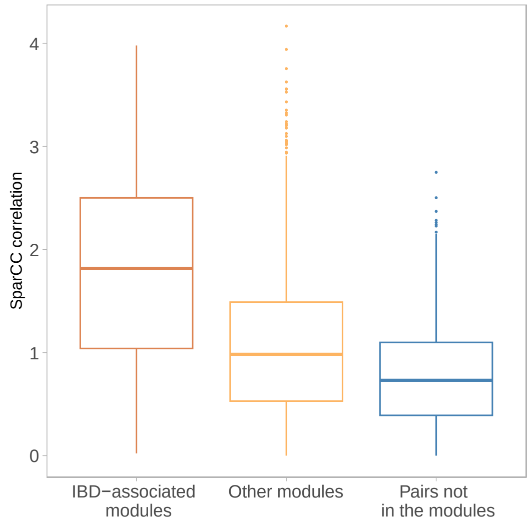

Supplementary Fig. S6. Illustration of pairwise correlation coefficients of taxa evaluated by SparCC. A. Heatmap demonstrating the correlation of each taxa pair. Taxa pairs in the IBD-associated modules are distributed in the green rectangle. **B.** Boxplot of correlation comparison of taxa pairs in the IBD-associated modules, other microbial modules and those not included in any module. The correlations are log-transformed after multiplying a scaling factor of 100.

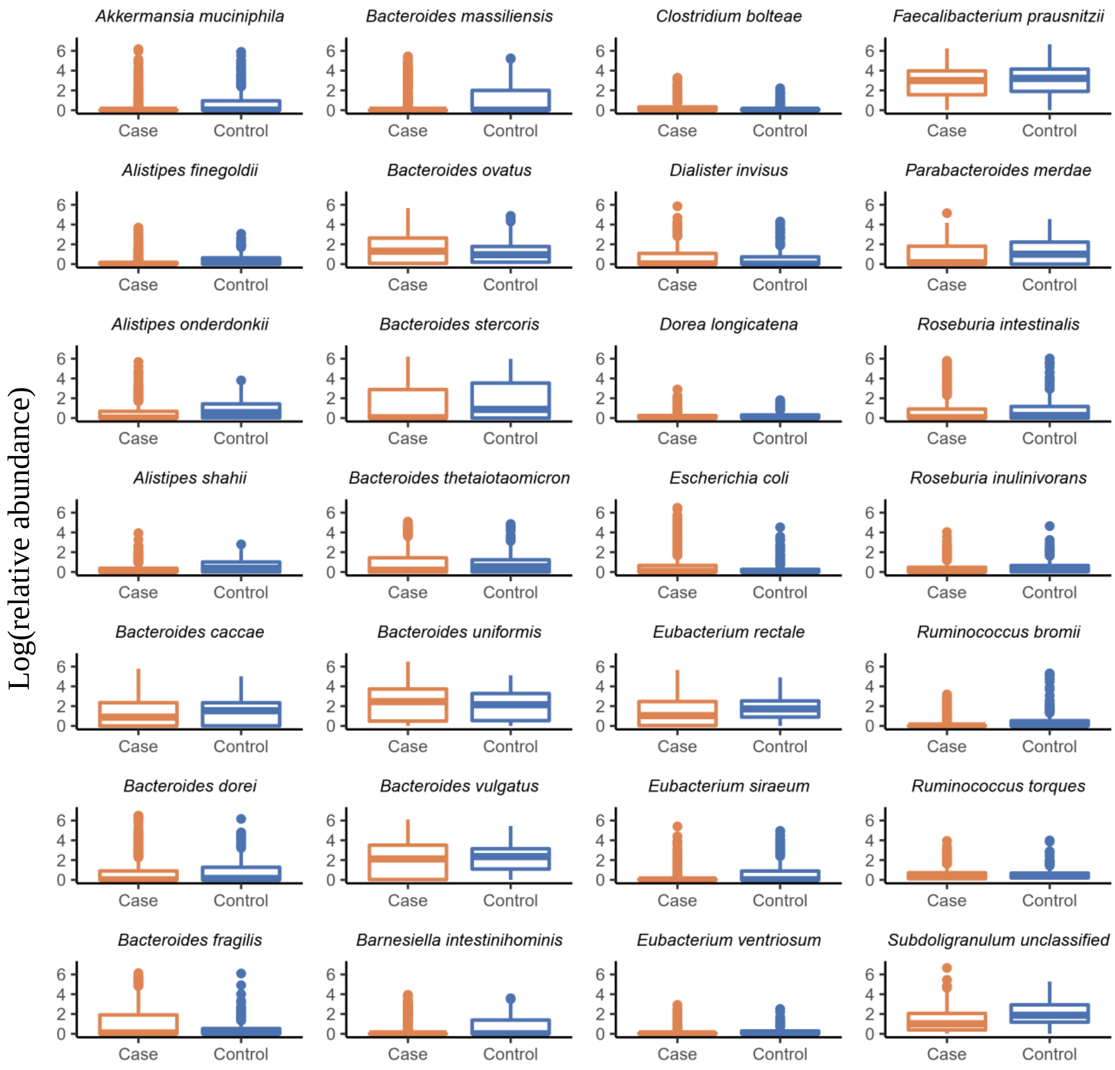

Supplementary Fig. S7. Relative abundance (log-transformed) of the taxa included in the IBD-associated modules in the IBD and control samples.

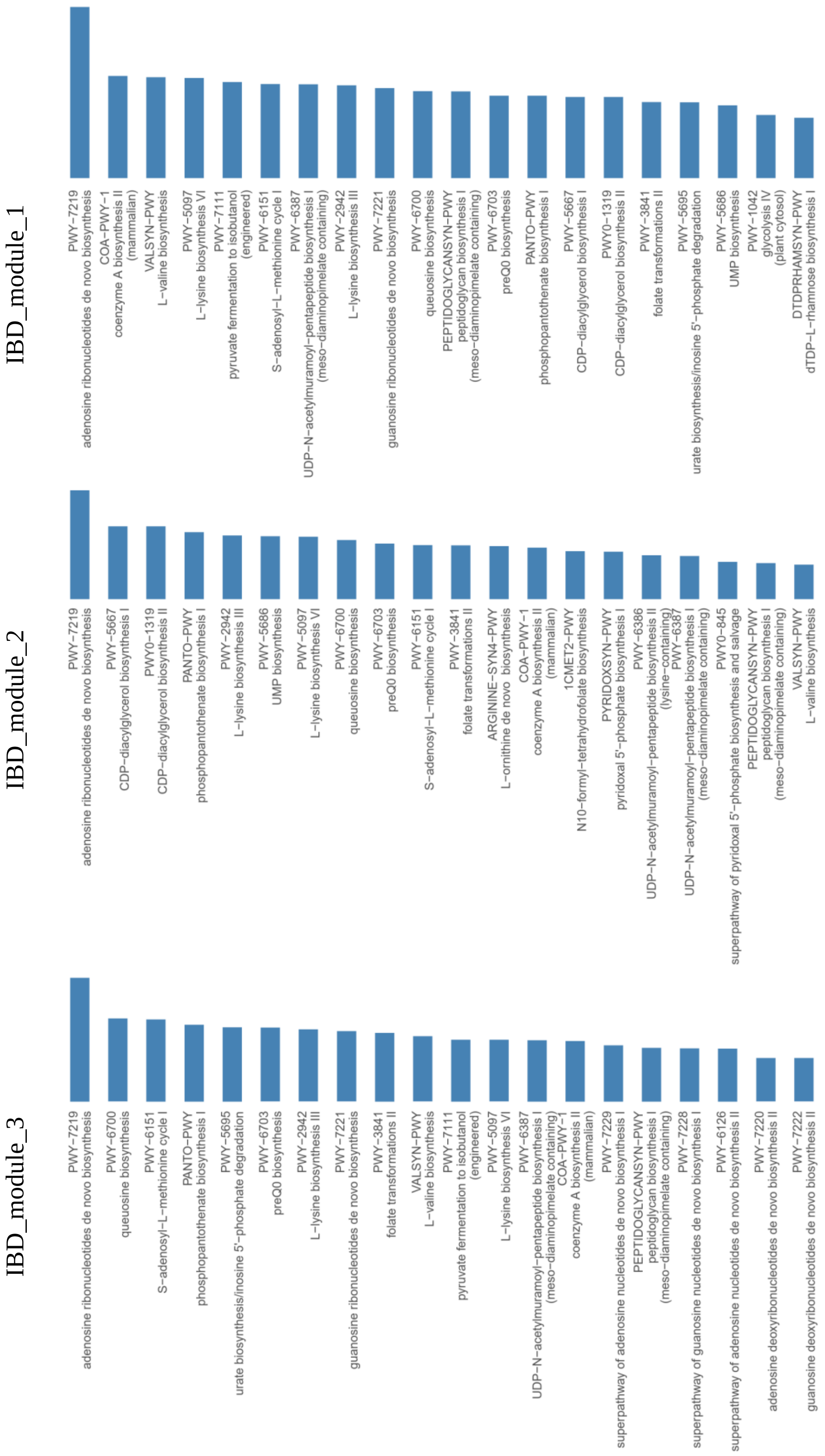

Supplementary Fig. S8. The abundance of the top 20 pathways in each IBD-associated module.

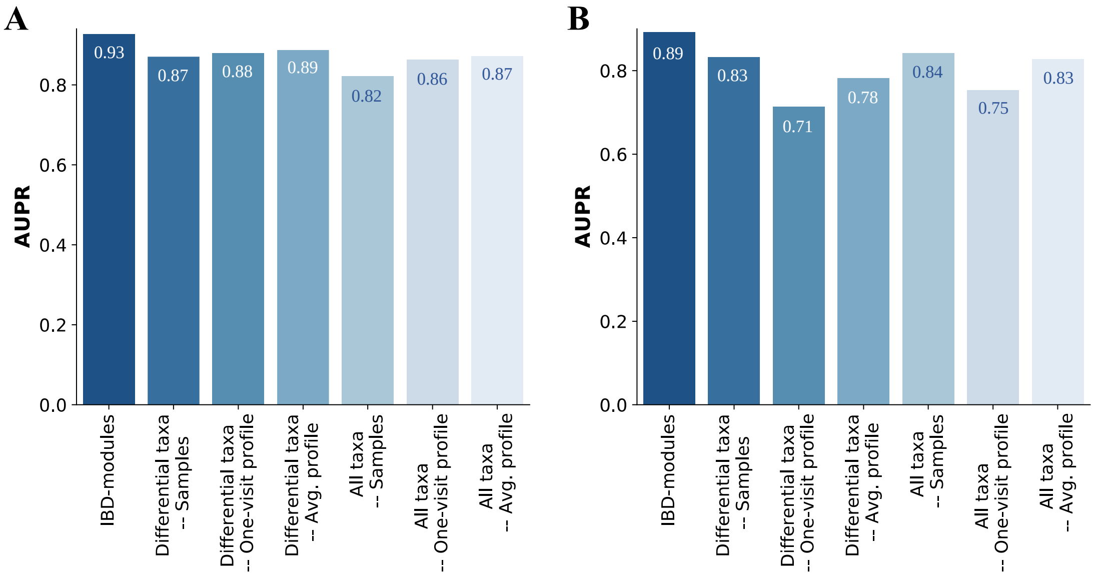

Supplementary Fig. S9. The classification performance (AUPR) of the IBD-associated modules and the taxa relative abundance in the discovery set (A) and the validation set *“Val_Hall”* (B), respectively.

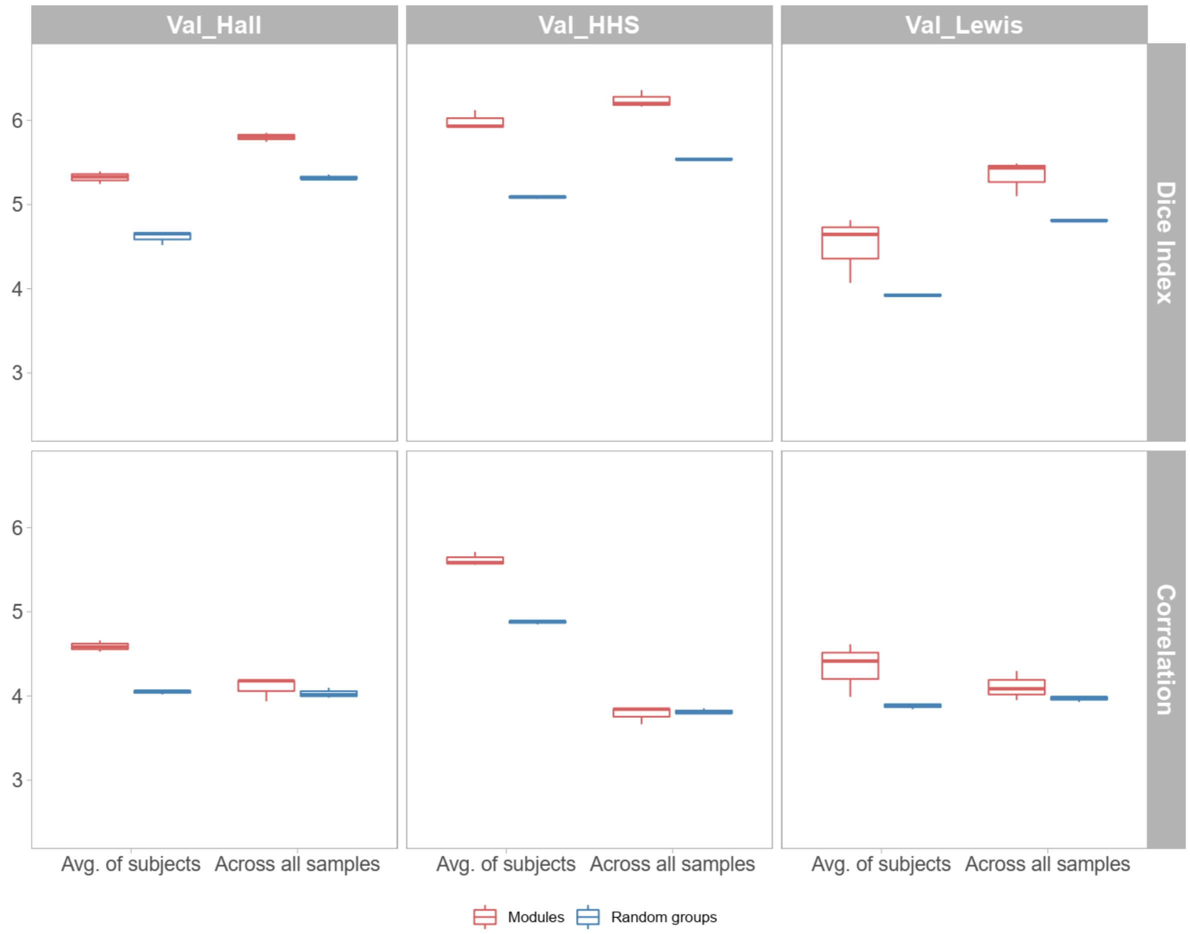

Supplementary Fig. S10. The intra-module Dice Index and absolute Spearman correlation of the IBD-associated modules in the validation cohorts.

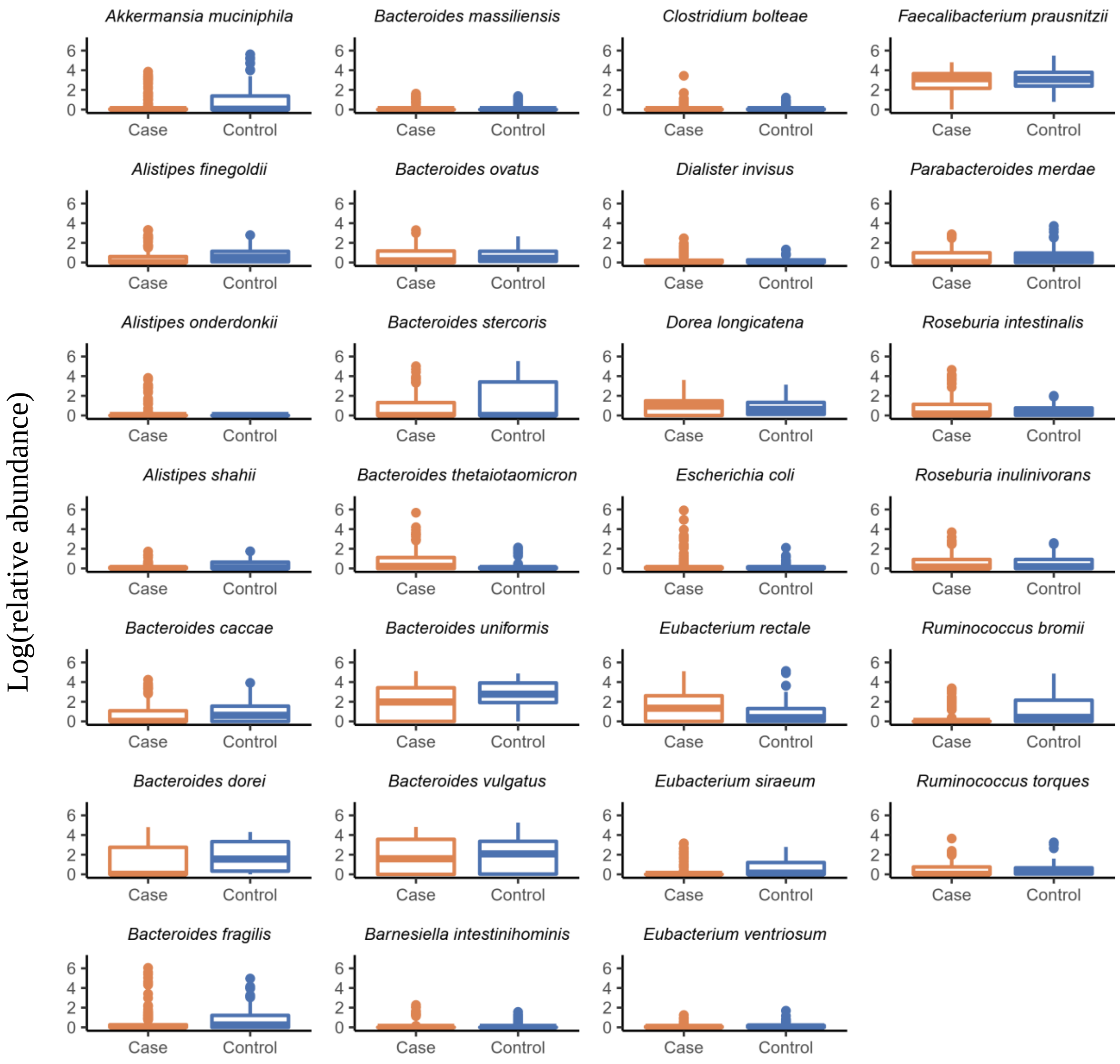

Supplementary Fig. S11. Relative abundance (log-transformed) of the taxa included in the IBD-associated modules in the IBD and control samples of the validation set *“Val_Hall”*.

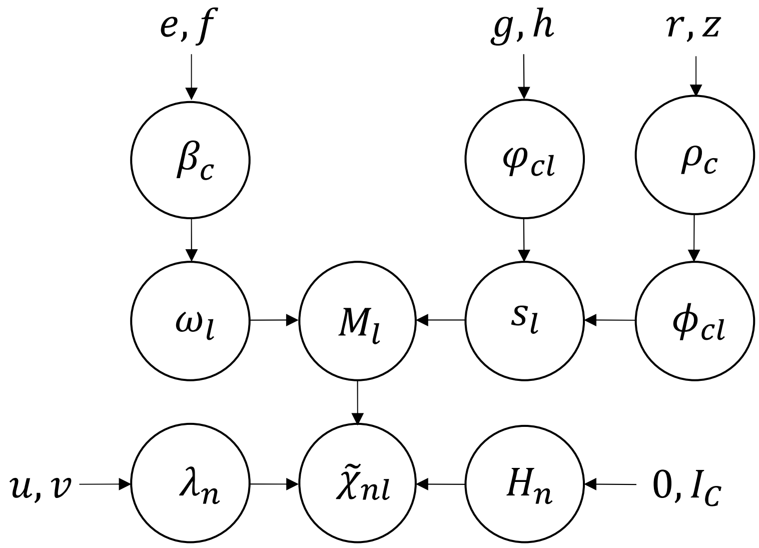

Supplementary Fig. S12. The Bayesian model of the 2D version of the proposed method to factorize the profile matrix.

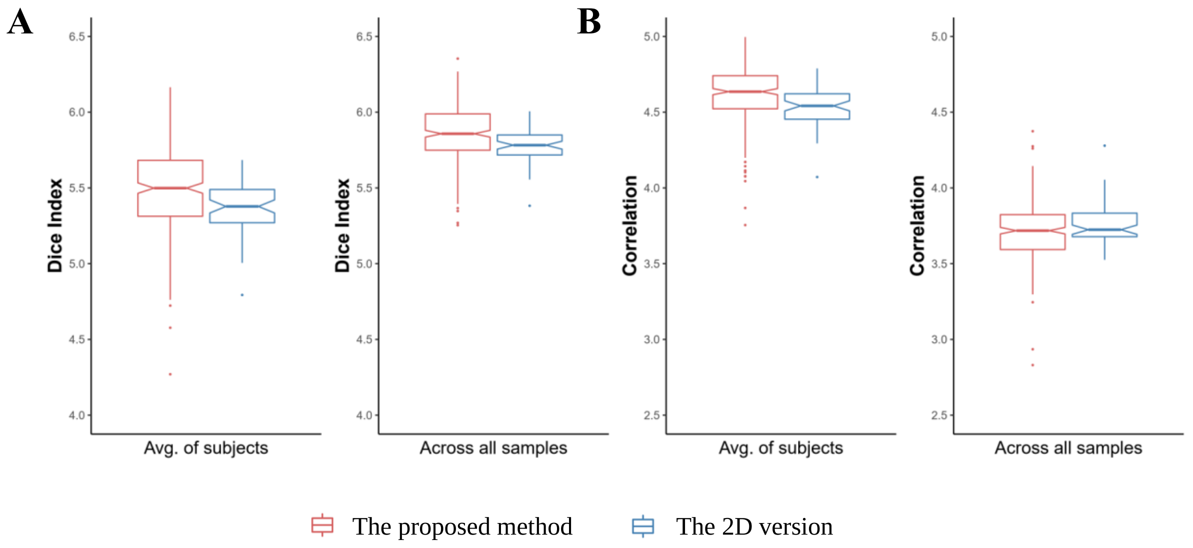

Supplementary Fig. S13. The intra-module Dice Index (A) and absolute Spearman correlation (B) of the microbial modules identified by the proposed method (red boxes) and the 2D version (blue boxes) in the discovery set.

Supplementary Tables

Supplementary Table S1. Clinical information statistics of the *"10-visit-set"*

		IBD		Control	All
		CD	UC		
**Subjects**		46	25	25	96
**Age**	Mean ± SD	25.91 ± 17.21	29.32 ± 18.97	29.72 ± 20.87	27.79 ± 18.56
	≤ 18	25 (54%)	11 (44%)	12 (48%)	48 (50%)
	19 - 30	6 (13%)	4 (16%)	3 (12%)	13 (14%)
	31 - 40	4 (9%)	3 (12%)	2 (8%)	9 (9%)
	41 - 50	5 (11%)	5 (20%)	1 (4%)	11 (11%)
	51 - 60	4 (9%)	0 (0%)	4 (16%)	8 (8%)
	> 60	2 (4%)	2 (8%)	3 (12%)	7 (7%)
**Sex**	Female	21 (46%)	16 (64%)	11 (44%)	48 (50%)
	Male	25 (54%)	9 (36%)	14 (56%)	48 (50%)
**Race**	American Indian or Alaska Native	1 (2%)	0 (0%)	0 (0%)	1 (1%)
	Black or African American	0 (0%)	3 (12%)	0 (0%)	3 (3%)
	White	41 (89%)	21 (84%)	24 (96%)	86 (90%)
	Other	2 (4%)	0 (0%)	0 (0%)	2 (2%)
	More than one race	2 (4%)	1 (4%)	1 (4%)	4 (4%)
**Medication**	Yes	39 (85%)	14 (56%)	10 (40%)	63 (66%)
	No	7 (15%)	11 (44%)	15 (60%)	33 (34%)
**Antibiotics**	Yes	26 (57%)	8 (32%)	9 (36%)	43 (45%)
	No	20 (43%)	17 (68%)	16 (64%)	53 (55%)
**Immuno-suppressant**	Yes	27 (59%)	9 (36%)	0 (0%)	36 (38%)
	No	19 (41%)	16 (64%)	25 (100%)	60 (62%)
**Chemotherapy**	Yes	9 (20%)	2 (8%)	1 (4%)	12 (13%)
	No	37 (80%)	23 (92%)	24 (96%)	84 (87%)
**Diarrhea**	Yes	41 (89%)	25 (100%)	20 (80%)	86 (90%)
	No	5 (11%)	0 (0%)	5 (20%)	10 (10%)
**Bowel surgery**	Yes	12 (26%)	2 (8%)	0 (0%)	14 (15%)
	No	34 (74%)	23 (92%)	25 (100%)	82 (85%)

Supplementary Table S2. Differential taxa between the IBD and control groups identified by DA analysis by LMM

Taxon	P-value (BH corrected)
Alistipes putredinis	0.0017
Alistipes shahii	0.0063
Eubacterium siraeum	0.0099
Ruminococcus bromii	0.0121
Ruminococcus callidus	0.0023
Subdoligranulum unclassified	0.0002
Escherichia unclassified	0.0150
Akkermansia muciniphila	0.0060

Supplementary Table S3. IBD-associated taxa collected from HMDAD

Taxon	Disease
Bacteroides fragilis	Ileal Crohn's disease (CD)
Bacteroides ovatus	Crohn's disease (CD)
Bacteroides uniformis	Crohn's disease (CD)
Bacteroides vulgatus	Crohn's disease (CD)
Clostridium leptum	Crohn's disease (CD)
Enterococcus faecium	Crohn's disease (CD)
Escherichia coli	Crohn's disease (CD)
Faecalibacterium prausnitzii	Crohn's disease (CD)

Supplementary Table S4. Differentially abundant taxa detected in Ma *et al.*, 2021

Taxon		
Bacteroides caccae	*Bacteroides vulgatus*	*Eubacterium eligens*
Bacteroides cellulosilyticus	*Bifidobacterium adolescentis*	*Eubacterium hallii*
Bacteroides coprocola	*Bifidobacterium bifidum*	*Eubacterium rectale*
Bacteroides fragilis	*Bifidobacterium longum*	*Eubacterium siraeum*
Bacteroides massiliensis	*Butyrivibrio crossotus*	*Faecalibacterium prausnitzii*
Bacteroides ovatus	*Clostridium bolteae*	*Parabacteroides distasonis*
Bacteroides plebeius	*Clostridium clostridioforme*	*Roseburia intestinalis*
Bacteroides sp_2_1_22	*Collinsella aerofaciens*	*Roseburia inulinivorans*
Bacteroides sp_4_3_47FAA	*Coprococcus comes*	*Ruminococcus bromii*
Bacteroides stercoris	*Coprococcus sp_ART55_1*	*Ruminococcus obeum*
Bacteroides thetaiotaomicron	*Dialister invisus*	*Ruminococcus sp_5_1_39BFAA*

Supplementary Table S5. Differentially abundant taxa detected in Franzosa *et al.*, 2019

Taxon		
Adlercreutzia equolifaciens	*Coprococcus comes*	*Lachnospiraceae bacterium_4_1_37FAA*
Alistipes finegoldii	*Dorea formicigenerans*	*Lachnospiraceae bacterium_5_1_63FAA*
Alistipes indistinctus	*Dorea longicatena*	*Lachnospiraceae bacterium_7_1_58FAA*
Alistipes onderdonkii	*Dorea unclassified*	*Lachnospiraceae bacterium_9_1_43BFAA*
Alistipes putredinis	*Enterococcus faecium*	*Lactobacillus gasseri*
Alistipes senegalensis	*Escherichia coli*	*Oscillibacter unclassified*
Alistipes shahii	*Escherichia unclassified*	*Pediococcus acidilactici*
Anaerostipes hadrus	*Eubacterium eligens*	*Pediococcus unclassified*
Anaerotruncus unclassified	*Eubacterium hallii*	*Roseburia hominis*
Bacteroidales bacterium ph8	*Eubacterium ramulus*	*Roseburia intestinalis*
Barnesiella intestinihominis	*Eubacterium rectale*	*Roseburia inulinivorans*
Bifidobacterium breve	*Eubacterium siraeum*	*Roseburia unclassified*
Blautia producta	*Eubacterium ventriosum*	*Ruminococcus callidus*
Clostridium clostridioforme	*Faecalibacterium prausnitzii*	*Ruminococcus gnavus*
Clostridium symbiosum	*Gordonibacter pamelaeae*	*Ruminococcus lactaris*
Coprococcus catus	*Holdemania filiformis*	*Ruminococcus obeum*
Ruminococcus sp_5_1_39BFAA	*Subdoligranulum unclassified*	

Supplementary Table S6. Other supports for taxa in the IBD-associated modules

Taxon	Support
Bacteroides dorei	Vidal *et al.*, 2015
Ruminococcus torques	Takahashi *et al.*, 2016
Parabacteroides merdae	Nomura *et al.*, 2021

Supplementary Table S7. Comparison of classification performance on the discovery set

		AUROC	AUPR	Sensitivity	Specificity	Precision
IBD-associated modules		0.80	0.93	0.74	0.97	0.99
Differential taxa	Samples	0.73	0.87	0.73	0.78	0.91
	One-visit profile	0.71	0.88	0.77	0.83	0.95
	Average profile	0.75	0.89	0.82	0.80	0.93
All taxa	Samples	0.62	0.82	0.63	0.77	0.91
	One-visit profile	0.69	0.86	0.77	0.80	0.93
	Average profile	0.72	0.87	0.72	0.83	0.94

Supplementary Table S8. Comparison of classification performance on the validation cohort *"Val_Hall"*

		AUROC	AUPR	Sensitivity	Specificity	Precision
IBD-associated modules		0.85	0.89	1.00	0.67	0.82
Differential taxa	Samples	0.71	0.83	0.69	0.79	0.88
	One-visit profile	0.65	0.71	0.72	0.73	0.80
	Average profile	0.66	0.78	0.72	0.67	0.76
All taxa	Samples	0.77	0.84	0.78	0.63	0.82
	One-visit profile	0.74	0.75	0.88	0.61	0.77
	Average profile	0.81	0.83	0.94	0.58	0.77

Supplementary Methods

Identification of gut microbial modules

We implemented two models in this study: the *visit-correlated model* (the proposed method) and the *visit-uncorrelated model* (as a comparison). The former accounted for the correlations between samples from the same subject and assumed dependent activities of the microbial modules in the same subject across different time points (**Fig. 2A**). The latter did not model the correlations and was applicable to both complete and incomplete tensor by incorporating an indicator matrix to specify the missing values (**Supplementary Fig. S2**).

1. Visit-correlated model

The full model of the *visit-correlated model* is as follows:

$$P(\tilde{\chi}|\theta) = \prod_{nlk} \mathcal{N}\left(\tilde{\chi}_{nlk} \middle| \sum_c H_{nc} M_{cl} T_{kc}, {\lambda_{nk}}^{-1}\right)$$

$$P(\lambda_{nk}) = Gamma(u, v)$$

$$P(H_{n.}) = \mathcal{N}_C(0, I_C)$$

$$M_{cl} = \omega_{cl} s_{cl}$$

$$P(\omega_{cl}|\beta_c) = \mathcal{N}\left(\omega_{cl}|0, {\beta_c}^{-1}\right)$$

$$P(\beta_c) = Gamma(\beta_c|e, f\,)$$

$$P(s_{cl}|\varphi_{cl}, \phi_{cl}) = Bernoulli(s_{cl}|\varphi_{cl}\phi_{cl})$$

$$P(\varphi_{cl}) = Beta(\varphi_{cl}|g, h\,)$$

$$P(\phi_{cl}|\rho_c) = Bernoulli(\phi_{cl}|\rho_c)$$

$$P(\rho_c) = Beta(\rho_c|r, z\,)$$

$$P\left(T_{kc}\middle|T_{k-1,c}, \Lambda_c\right) = \mathcal{N}\left(T_{k-1,c}, {\Lambda_c}^{-1}\,\right) \;\; (k = 2, \cdots, K)$$

$$P\left(T_{1,c}\middle|\Lambda_c\right) = \mathcal{N}\left(0, {\Lambda_c}^{-1}\right)$$

$$P(\Lambda_c) = Gamma(a, b)$$

We used Variational Bayes (VB) (Jordan *et al.*, 1999) to estimate the posterior distributions due to the large size of the relative abundance tensor. VB provides approximated results but is much faster than the alternative method Markov Chain Monte Carlo (MCMC). VB aims to find the setting of the parameters of a selected family of distributions $Q(\Theta)$ that is closest to the posterior distribution by minimizing the Kullback–Leibler (KL) divergence between $Q(\Theta)$ and $P(\theta|\tilde{\chi})$. The distribution of $Q(\theta)$ is the form of

$$Q(\theta) = \prod_{n,c} Q(H_{nc}) \prod_{k,c} Q(T_{kc}) \prod_{c} Q(\Lambda_c) \prod_{c,l} Q(\omega_{cl}|s_{cl}) Q(s_{cl}) \prod_{c} Q(\beta_c)$$
$$\prod_{c,l} \delta_{\varphi_{cl}^*}(\varphi_{cl}) \prod_{c,l} \delta_{\phi_{cl}^*}(\phi_{cl}) \prod_{c} \delta_{\rho_c^*}(\rho_c) \prod_{n,k} Q(\lambda_{nk})$$

where $\delta(x)$ is the Dirac delta function. Minimizing the KL-divergence between $Q(\theta)$ and $P(\theta|\tilde{\chi})$ is equivalent to maximizing the evidence lower bound (ELBO):

$$ELBO = E_Q[\log P(\tilde{\chi}, \theta)] - E_Q[\log Q(\theta)]$$
$$= \frac{1}{2} L \sum_{n,k} \langle \log \lambda_{nk} \rangle - \frac{1}{2} \sum_{n,l,k} \langle \lambda_{nk} \rangle \left\langle \left(\tilde{\chi}_{nlk} - \sum_c \langle H_{nc} \rangle \langle s_{cl} w_{cl} \rangle \langle T_{kc} \rangle \right)^2 \right\rangle$$

$$-\frac{1}{2}\sum\nolimits_{n,c}\langle {H_{nc}}^2\rangle - \frac{1}{2}\sum\nolimits_{n,c}\log|\Omega_{nc}^*|$$

$$-\frac{1}{2}\sum\nolimits_{c,k\in\{2:k\}}\langle \Lambda_c\rangle \langle (T_{kc}-T_{k-1,c})^2\rangle - \frac{1}{2}\sum\nolimits_{c}\langle \Lambda_c\rangle\langle {T_{1,c}}^2\rangle + \frac{1}{2}\sum\nolimits_{k,c}\log p_{kc}^*$$

$$+\sum\nolimits_{c}\left[\left(a - a_c^* + \frac{1}{2}T\right)\langle \log \Lambda_c\rangle + a_c^*\left(1+\log|b_c^*| + \frac{b_c^*}{b}\right) + \log\Gamma(a_c^*)\right]$$

$$+\sum\nolimits_{n,k}\left[(u-1)\langle \log\lambda_{nk}\rangle - \frac{1}{v}\langle \lambda_{nk}\rangle\right]$$

$$-\sum\nolimits_{n,k}[(u_{nk}^*-1)\langle \log\lambda_{nk}\rangle - u_{nk}^*(1+\log|v_{nk}^*|) - \log\Gamma(u_{nk}^*)]$$

$$+\frac{1}{2}\sum\nolimits_{c}\langle \log\beta_c\rangle - \frac{1}{2}\sum\nolimits_{c,l}\langle \beta_c\rangle\langle {w_{cl}}^2\rangle$$

$$-\frac{1}{2}\sum\nolimits_{c,l}\gamma_{cl}^* log(\sigma_{cl}^*) + \frac{1}{2}\sum\nolimits_{c,l}(1-\gamma_{cl}^*)\langle \log\beta_c\rangle$$

$$+\sum\nolimits_{c}(e-1)\langle \log\beta_c\rangle - \sum\nolimits_{c}\frac{1}{f}\langle \beta_c\rangle - \sum\nolimits_{c}[(e_c^*-1)\langle \log\beta_c\rangle - e_c^* - \log\Gamma(e_c^*)]$$

$$+\sum\nolimits_{c,l}[\gamma_{cl}^* log(\varphi_{cl}^*\phi_{cl}^*) + (1-\gamma_{cl}^*)\log(1-\varphi_{cl}^*\phi_{cl}^*) - \gamma_{cl}^*\log\gamma_{cl}^* - (1-\gamma_{cl}^*)\log(1-\gamma_{cl}^*)]$$

$$+\sum\nolimits_{c,l}[(g-1)\log\varphi_{cl}^* + (h-1)\log(1-\varphi_{cl}^*)]$$

$$+\sum\nolimits_{c,l}[\phi_{cl}^*\log\rho_c^* + (1-\phi_{cl}^*)\log(1-\rho_c^*)]$$

$$+\sum\nolimits_{c}[(r-1)\log\rho_c^* + (z-1)\log(1-\rho_c^*)]$$

The updates for φ_{cl}, ϕ_{cl} and ρ_c were done by point estimation (Hore *et al.*, 2016). The updates for the other variables are as follows:

- Factor matrix H:

$$Q(H_{nc}) = \mathcal{N}\left(H_{nc}\middle|\mu_{nc}^*, {\Omega_{nc}^*}^{-1}\right)$$
$$\Omega_{nc}^* = 1 + \sum\nolimits_{l,k}\langle \lambda_{nk}\rangle\langle {s_{cl}}^2{w_{cl}}^2\rangle\langle {T_{kc}}^2\rangle$$
$$\mu_{nc}^* = {\Omega_{nc}^*}^{-1}\left(\sum\nolimits_{l,k}\widetilde{\chi}_{nlk}\langle \lambda_{nk}\rangle\langle s_{cl}w_{cl}\rangle\langle T_{kc}\rangle - \sum\nolimits_{l,k}\langle \lambda_{nk}\rangle\langle s_{cl}w_{cl}\rangle\langle T_{kc}\rangle\sum\nolimits_{m\neq c}\langle H_{nm}\rangle\langle s_{ml}w_{ml}\rangle\langle T_{km}\rangle\right)$$

- Factor matrix T and precision Λ:

$$Q(T_{kc}) = \mathcal{N}\left(T_{kc}\middle|o_{kc}^*, {p_{kc}^*}^{-1}\right)$$
$$o_{kc}^* = \sum\nolimits_{n,l}\langle \lambda_{nk}\rangle\langle {H_{nc}}^2\rangle\langle {s_{cl}}^2{w_{cl}}^2\rangle + Z_{kc}$$

$$p_{kc}^{*} = {o_{kc}^{*}}^{-1}\left(\sum\nolimits_{n,l}\widetilde{\mathcal{X}}_{nlk}\langle\lambda_{nk}\rangle\langle H_{nc}\rangle\langle s_{cl}w_{cl}\rangle - \sum\nolimits_{n,l}\langle\lambda_{nk}\rangle\langle H_{nc}\rangle\langle s_{cl}w_{cl}\rangle\sum\nolimits_{m\neq c}\langle H_{nm}\rangle\langle s_{ml}w_{ml}\rangle\langle T_{km}\rangle + Y_{kc}\right)$$

$$Z_{kc} = \begin{cases} 2\langle\Lambda_c\rangle, if\ k < K \\ \langle\Lambda_c\rangle, if\ k = K \end{cases}$$

$$Y_{kc} = \begin{cases} \langle\Lambda_c\rangle\langle T_{2,c}\rangle, & if\ k = 1 \\ \langle\Lambda_c\rangle\left(\langle T_{k-1,c}\rangle + \langle T_{k+1,c}\rangle\right), & if\ 1 < k < K \\ \langle\Lambda_c\rangle\langle T_{K-1,c}\rangle, & if\ k = K \end{cases}$$

$$Q(\Lambda_c) = \mathcal{G}(\Lambda_c|a_c^{*}, b_c^{*})$$

$$a_c^{*} = a + \frac{1}{2}K$$

$$b_c^{*} = \begin{cases} \left(\frac{1}{b} + \frac{1}{2}\langle {T_{1,c}}^2\rangle\right)^{-1}, & if\ k = 1 \\ \left(\frac{1}{b} + \frac{1}{2}\sum\nolimits_{k}\langle\left(T_{k,c} - T_{k-1,c}\right)^2\rangle\right)^{-1}, & otherwise \end{cases}$$

- Sparsity parameters of factor matrix M:

$$Q(w_{cl}|s_{cl}) = \mathcal{N}(w_{cl}|s_{cl}m_{cl}^{*}, (s_{cl}\sigma_{cl}^{*} + (1 - s_{cl})\langle\beta_c\rangle)^{-1})$$

$$\sigma_{cl}^{*} = \langle\beta_c\rangle + \sum\nolimits_{n,k}\langle\lambda_{nk}\rangle\langle {H_{nc}}^2\rangle\langle {T_{kc}}^2\rangle$$

$$m_{cl}^{*} = {\sigma_{cl}^{*}}^{-1}\left(\sum\nolimits_{n,k}\widetilde{\mathcal{X}}_{nlk}\langle\lambda_{nk}\rangle\langle H_{nc}\rangle\langle T_{kc}\rangle - \sum\nolimits_{k}\langle\lambda_{nk}\rangle\langle H_{nc}\rangle\langle T_{kc}\rangle\sum\nolimits_{m\neq c}\langle H_{nm}\rangle\langle s_{ml}w_{ml}\rangle\langle T_{km}\rangle\right)$$

$$Q(s_{cl}) = Bernoulli(s_{cl}|\gamma_{cl}^{*})$$

$$\gamma_{cl}^{*} = 1/\left(1 + e^{-\eta_{cl}^{*}}\right)$$

$$\eta_{cl}^{*} = log(\varphi_{cl}^{*}\phi_{cl}^{*}) - \frac{1}{2}log(\sigma_{cl}^{*}) + \frac{1}{2}\sigma_{cl}^{*}{m_{cl}^{*}}^2 - log(1 - \varphi_{cl}^{*}\phi_{cl}^{*}) + \frac{1}{2}log\langle\beta_c\rangle$$

$$Q(\beta_c) = \mathcal{G}(\beta_c|e_c^{*}, f_c^{*})$$

$$e_c^{*} = e + \frac{1}{2}L$$

$$f_c^{*} = \left(\frac{1}{f} + \frac{1}{2}\sum\nolimits_{l}\langle {w_{cl}}^2\rangle\right)^{-1}$$

- Noise term λ:

$$Q(\lambda_{nk}) = \mathcal{G}(\lambda_{nk}|u_{nk}^{*}, v_{nk}^{*})$$

$$u_{nk}^{*} = u + \frac{1}{2}L$$

$$v_{nk}^* = \left(\frac{1}{v} + \frac{1}{2}\sum_l \left\langle \left(\tilde{\chi}_{nlk} - \sum_c \langle H_{nc}\rangle\langle s_{cl}w_{cl}\rangle\langle T_{kc}\rangle\right)^2 \right\rangle\right)^{-1}$$

In each iteration, the algorithm updates the variables one by one and calculates the ELBO. In addition, $\langle S\rangle$ is calculated to check the convergence. The algorithm will continue to update the variables until the average changes of the modules drop to less than 1 taxa in the last ten iterations (Hore *et al.*, 2016). The maximum number of iterations was set to 2000.

After posterior estimation, the members of a module were determined by the posterior inclusion probabilities (PIP), calculated as $E_Q(s_{cl})$, which indicated the probability that a variable was included in the true model (Hore *et al.*, 2016). A taxon was determined to be a member of a module if the corresponding $PIP > 0.5$. The maximum number of modules C_{max} in the experiments was set to 300, because some preliminary results demonstrated that 300 was large enough for the underlying modules. Distinct initializations of the variables would lead to different factorization results. To find a better result and to conduct a fair evaluation of the overall performance of the model, 30 runs with random initializations were executed, except φ_{cl}, ϕ_{cl} and s_{cl}, which were initialized to 0.5. The result of each run was evaluated by the subject classification performance using the top-ranking modules with associations to IBD sorted by the Wilcoxon rank-sum test, where the modules with the lowest adjusted p-values (Benjamini–Hochberg method (Benjamini and Hochberg, 1995)) were selected.

2. Visit-uncorrelated model

For the *visit-uncorrelated model*, the activities of the modules in the samples were assumed to be independent $T_{k.} \sim \mathcal{N}_C(0, I_C)$. It was extended to be applicable to both complete and incomplete tensors by incorporating an indicator matrix P to mark the missing data:

$$P(\chi|\theta) = \prod_{n,l,k} \mathcal{N}\left(\chi_{nlk} \middle| \sum_c H_{nc}M_{cl}T_{kc}, {\lambda_{nk}}^{-1}\right)^{P_{nk}}$$

where $\chi = \{\tilde{\chi}, \hat{\chi}\}$. $\hat{\chi} \in \mathbb{R}^{97\times123\times24}$ was the incomplete tensor constructed from the *"24-visit-set"* where the relative abundance of the samples from each subject was placed in the cube according to their collected time points, leaving the unfilled entries as missing data. $P_{nk} = 1$ if the sample of subject n at time point k was collected in χ; otherwise, $P_{nk} = 0$. The updates with respect to T are as follows:

$$Q(T_{kc}) = \mathcal{N}\left(T_{kc} \middle| o_{kc}^*, {p_{kc}^*}^{-1}\right)$$

$$o_{kc}^* = \sum_{n,l} P_{nk}\langle\lambda_{nk}\rangle\langle {H_{nc}}^2\rangle\langle {s_{cl}}^2 {w_{cl}}^2\rangle$$

$$p_{kc}^* = {o_{kc}^*}^{-1}\left(\sum_{n,l} P_{nk}\chi_{nlk}\langle\lambda_{nk}\rangle\langle H_{nc}\rangle\langle s_{cl}w_{cl}\rangle - \sum_{n,l} P_{nk}\langle\lambda_{nk}\rangle\langle H_{nc}\rangle\langle s_{cl}w_{cl}\rangle \sum_{m\neq c}\langle H_{nm}\rangle\langle s_{ml}w_{ml}\rangle\langle T_{km}\rangle\right)$$

The updates for the other variables and the ELBO were also modified by adding the indicator matrix to summations over n and k in the same way as T_{kc}. The part regarding the factor matrix T in ELBO (the fourth and fifth lines) was replaced by the following expression:

$$-\frac{1}{2}\sum\nolimits_{kc}\langle T_{kc}{}^2\rangle-\frac{1}{2}\sum\nolimits_{kc}\log|p^*_{kc}|$$

The *visit-uncorrelated model* was applied to both *"10-visit-set"* ($\tilde{\chi}$) and *"24-visit-set"* ($\hat{\chi}$) and was inferred and evaluated in the same way as the *visit-correlated model*.

Relationships between the microbial taxa in the modules

To study the relationships between the microbial taxa in the same module, the intra-module co-occurrence and functional similarity were investigated, where the co-occurrence was measured by Dice Index (DI) and Spearman correlation, and the functional similarity was measured based on the contributed pathway abundance. Specifically, for each module, the members were identified by the PIP described above. For each pair of taxa (l, l') in the module, the pairwise DI and absolute Spearman correlation were computed (1) individually by $\frac{1}{N}\sum_n|C(\tilde{\chi}_{nl.},\tilde{\chi}_{nl'.})|$ and (2) across subjects by $|C(vec(\tilde{\chi}_{.l.}), vec(\tilde{\chi}_{.l'.}))|$, where $C(x, x')$ was the DI $(2|x \cap x'|/(|x| + |x'|))$ or Spearman correlation between x and x', and $vec(x)$ is the flattened vector of x. For the former, the co-occurrence was first calculated across the samples for each subject and averaged over all subjects afterwards to indicate the co-occurrence of the taxa over time. The latter measured the co-occurrence across the samples from all subjects regardless of the effects of subjects and sampling time, taking each sample as an independent community. Then, the overall DI and Spearman correlation for the module were calculated by averaging the above pairwise measures within the module, respectively. The functional similarity of a pair of taxa in a particular sample was evaluated by the Jaccard similarity of their contributions to the pathways based on the functional profile. The intra-module functional similarity was computed by averaging the pairwise similarity of member taxa over all samples. When demonstrated in the figures, all of these measures were first multiplied by a scaling factor of 100 and then log-transformed.

In addition, the co-occurrence and functional similarity of the identified microbial modules were compared to that of randomly generated microbial groups. The pairs of taxa not included in any modules were selected as the candidates to form the random groups. For each identified module, ten equal-size groups (groups consisting of an equal number of taxa pairs as the module) were generated by randomly selecting taxa pairs from the candidates. The intra-group DI was calculated for each of the ten groups in the same way as the modules and then averaged as the final comparison to the DI of the module. The Spearman correlation and functional similarity of the random groups were measured similarly.

Permutational multivariate analysis of variance (PERMANOVA)

The variation of taxonomic composition and the microbial module activities explained by several clinical factors, including disease states, medication, antibiotics, immunosuppressant, chemotherapy, diarrhea, bowel surgery, age, sex and race, were quantified via PERMANOVA using the adonis function in the R package "Vegan" (Oksanen *et al.*, 2020), respectively. The distances between the taxonomic composition of the samples were measured by Bray–Curtis dissimilarity. To address the issue of repeated measures of longitudinal data, restricted permutations were used in PERMANOVA. For the *sample-level factors*, including medication, antibiotics, immunosuppressant, diarrhea and bowel surgery, which recorded the status of the

subjects in the week of sample collection, blocked permutations within subjects were adopted by using the “strata” option of the adonis function. For the *subject-level factors*, including disease states, age, sex and race, which were constant across the samples of a subject, permutations of plots (i.e., groups of samples collected from the same subject) were performed. The subject “C3031” was removed from the analysis due to the missing age information.

Regular PERMANOVA was conducted to evaluate the variation of microbial modules explained by the factors at the subject-level, because the activities of the microbial modules were estimated with respect to each subject without the effects of repeated measures. For each sample-level factor, an overall value was required for each subject. It was determined by merging the values of the factor across the samples from the subject. Since the sample-level factors were binary valued, the overall value of a sample-level factor for a subject was labelled as positive (“Yes”) if there was at least one sample demonstrated positive for the factor; otherwise, it was labelled as negative (or “No”). For example, if a subject was recorded using antibiotics (positive) in at least one sample, the overall value of the factor for the subject was marked positive. Euclidean distance between the activity vectors of the modules, i.e., the rows of the factor matrix H, was used to measure the distances between the subjects.

IBD-associated microbial taxa

The IBD-associated microbial taxa were identified by (1) differential abundance (DA) analysis, (2) collecting microbe-disease associations from Human Microbe-Disease Association Database (HMDAD) (Ma *et al.*, 2017) and (3) collecting the IBD-associated taxa from other studies, and were listed in **Supplementary Tables S2-S6**.

Differentially abundant microbial taxa in terms of disease states (IBD and control) were identified from the log-transformed relative abundance profile consisting of all samples in the *“10-visit-set”*. The linear mixed-effects model (LMM) was used to account for the effects of repeated measures (Lloyd-Price *et al.*, 2019):

$$Relative\ abundance \sim (intercept) + disease\ states + age + \ antibiotics\ \ + 1|subject$$

where the relative abundance of each taxon was modelled as a regression of the intercept, disease states, age and antibiotics use with subjects as a random variable. It was fitted using the lme function in the R package “nlme” (DebRoy, 2006). The estimated coefficient of disease states with respect to each taxon was used to indicate the association between them. Differentially abundant taxa were then identified with a target FDR of 0.25 (Lloyd-Price *et al.*, 2019).

HMDAD is a resource that integrates the microbe-disease associations discovered in previous studies. Microbial associations in HMDAD were downloaded from http://www.cuilab.cn/hmdad. The associations of IBD, UC and CD at species level were selected, where the associated species not included in the discovery set were removed. In addition, we also collected the differentially abundant taxa detected in another two studies of IBD (Franzosa *et al.*, 2019; Ma *et al.*, 2021).

Classification of the subjects using relative abundance

In comparison to microbial modules, we also evaluated the classification performance of the relative abundance of the differentially abundant taxa as well as all microbial taxa. RF with 2000 trees was used and tuned by the R package caret (Kuhn, 2015). To thoroughly investigate

the classification performance of relative abundance, we trained and tested RF models on the samples, the averaged profile and randomly generated one-visit profiles of the *"10-visit-set"*, respectively. For sample classification, the profile containing all samples was used. The averaged profile was calculated by averaging the samples collected from the same subject, characterizing each subject by the averaged relative abundance of taxa over time. We conducted 10-fold cross-validation and calculated the average AUROC over the 10 folds for the all-sample profile and the averaged profile, respectively. For the evaluation of one-visit profiles, we generated ten one-visit profiles where each one was created by randomly selecting one sample from each subject. We calculated the average AUROC of 10-fold cross-validation for each one-visit profile and then evaluated the overall performance by averaging AUROC over the 10 profiles. The corresponding AUPR, sensitivity, specificity and precision were calculated similarly.

References

Benjamini, Y. and Hochberg, Y. (1995) 'Controlling the false discovery rate: a practical and powerful approach to multiple testing', *Journal of the Royal statistical society: series B (Methodological)*, 57(1), pp. 289–300.

DebRoy, S. (2006) 'NLME: Linear and nonlinear mixed effects models', *R package* [Preprint].

Franzosa, E.A. *et al.* (2019) 'Gut microbiome structure and metabolic activity in inflammatory bowel disease', *Nature microbiology*, 4(2), pp. 293–305.

Hore, V. *et al.* (2016) 'Tensor decomposition for multiple-tissue gene expression experiments', *Nature genetics*, 48(9), pp. 1094–1100.

Jordan, M.I. *et al.* (1999) 'An introduction to variational methods for graphical models', *Machine learning*, 37(2), pp. 183–233.

Kuhn, M. (2015) 'Caret: classification and regression training', *Astrophysics Source Code Library*, p. ascl--1505.

Lloyd-Price, J. *et al.* (2019) 'Multi-omics of the gut microbial ecosystem in inflammatory bowel diseases', *Nature*, 569(7758), pp. 655–662.

Ma, W. *et al.* (2017) 'An analysis of human microbe--disease associations', *Briefings in bioinformatics*, 18(1), pp. 85–97.

Ma, Y. *et al.* (2021) 'Metagenome Analysis of Intestinal Bacteria in Healthy People, Patients With Inflammatory Bowel Disease and Colorectal Cancer', *Frontiers in Cellular and Infection Microbiology*, 11.

Oksanen, J. *et al.* (2020) 'vegan: Community Ecology Package'.
